# Reconstruction of the Fas-Based Death-Inducing Signaling Complex (DISC) Using a Protein–Protein Docking Meta-Approach

**DOI:** 10.1021/acs.jcim.1c00301

**Published:** 2021-07-01

**Authors:** Sayyed Jalil Mahdizadeh, Melissa Thomas, Leif A. Eriksson

**Affiliations:** Department of Chemistry and Molecular Biology, University of Gothenburg, 405 30 Göteborg, Sweden

## Abstract

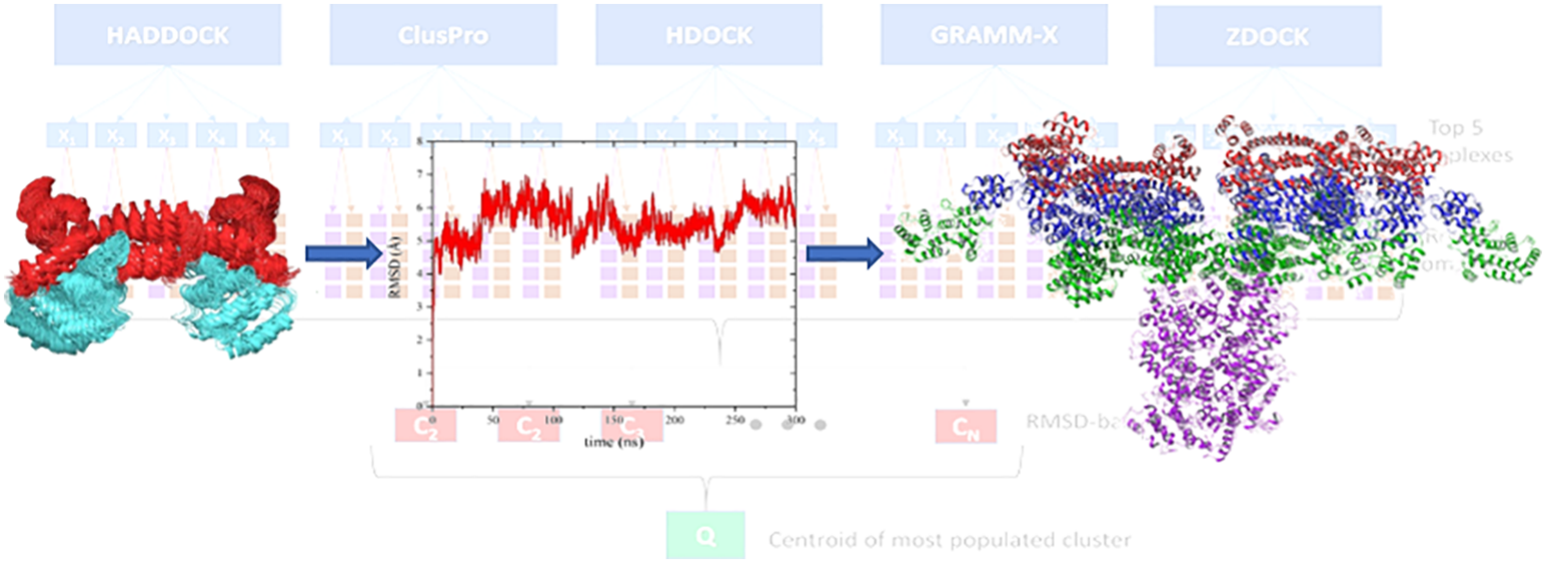

The death-inducing signaling complex (DISC) is a fundamental multiprotein complex, which triggers the extrinsic apoptosis pathway through stimulation by death ligands. DISC consists of different death domain (DD) and death effector domain (DED) containing proteins such as the death receptor Fas (CD95) in complex with FADD, procaspase-8, and cFLIP. Despite many experimental and theoretical studies in this area, there is no global agreement neither on the DISC architecture nor on the mechanism of action of the involved species. In the current work, we have tried to reconstruct the DISC structure by identifying key protein interactions using a new protein–protein docking meta-approach. We combined the benefits of five of the most employed protein–protein docking engines, HADDOCK, ClusPro, HDOCK, GRAMM-X, and ZDOCK, in order to improve the accuracy of the predicted docking complexes. Free energy of binding and hot spot interacting residues were calculated and determined for each protein–protein interaction using molecular mechanics generalized Born surface area and alanine scanning techniques, respectively. In addition, a series of *in-cellulo* protein-fragment complementation assays were conducted to validate the protein–protein docking procedure. The results show that the DISC formation initiates by dimerization of adjacent Fas_DD_ trimers followed by recruitment of FADD through homotypic DD interactions with the oligomerized death receptor. Furthermore, the *in-silico* outcomes indicate that cFLIP cannot bind directly to FADD; instead, cFLIP recruitment to the DISC is a hierarchical and cooperative process where FADD initially recruits procaspase-8, which in turn recruits and heterodimerizes with cFLIP. Finally, a possible structure of the entire DISC is proposed based on the docking results.

## Introduction

Proteins play a principal role in many essential biological processes within the living organisms, ranging from signal transduction and enzyme catalysis to gene expression and metabolism. However, proteins rarely perform their *in vivo* tasks as isolated species; instead, they interact with other proteins and other biomolecules such as RNA and DNA in sophisticated “molecular networks”. It has been demonstrated that more than 80% of all proteins are involved in at least one protein–protein interaction (PPI).^1^ It is estimated that there are 600 000 different PPIs in the human interactome^2,3^ which exceeds the number of proteins in the proteome by one order of magnitude.^4^ PPIs are thus as important as the proteins themselves for cell survival.^5^ Moreover, a profound understanding of PPIs and identifying the related key interacting residues is necessary in order to design drug molecules which can interfere with specific pathways as novel therapeutic disease intervention.^6^

**Figure 1:**
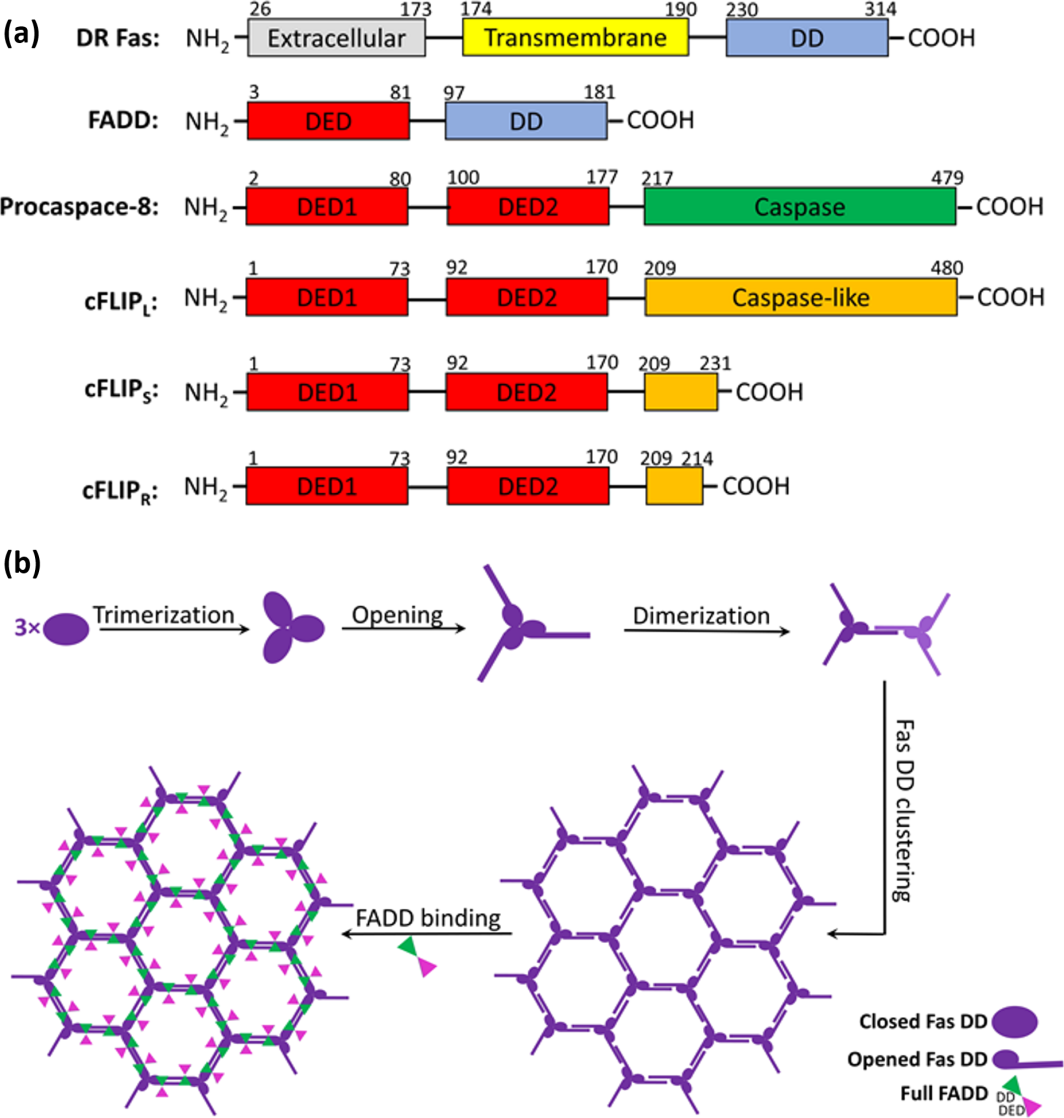
(a) DD and DED containing proteins involved in the Fas-induced DISC formation. Numbers indicate the starting and ending residues of each subdomain. (b) Model illustrating the proposed Fas_DD_–Fas_DD_ bridge and Fas_DD_–FADD_full_ formation mechanism. As a result of DL stimulation, Fas_DDs_ form homotrimeric complexes. The Fas_DD_ opening allows the formation of an extensive network by dimerization of Fas_DD_ (through the stem helices) in adjacent trimeric Fas_DDs_. Subsequently, FADD molecules are recruited to the Fas network through homotypic DD interactions.

One such system, which relies on a large number of protein–protein interactions is the death-inducing signaling complex (DISC). DISC formation is the earliest stage in the extrinsic apoptosis signaling pathway. It forms after stimulation of the extracellular domain of death receptors (DRs), here Fas (CD95), by death ligands (DLs), which subsequently triggers the programed cell death. DISC consists of different death domain (DD) and death effector domain (DED) containing proteins (Figure 1a) such as the intercellular part of DR, the adaptor protein Fas-associated death domain (FADD), procaspase 8 (C8), and its inhibitor FLICE-like inhibitory protein (cFLIP_L,S,R_).^7^

At the first stage of DISC formation, the cytosolic DD of the death receptors (Fas in Figure 1a) trimerize and oligomerize as a consequence of stimulation by death ligands (DL).^8,9^ Subsequently, FADD is recruited to the DISC through homotypic DD interactions with the oligomerized DR (Figure 1b). At the next step, C8 and/or cFLIP add to the DISC through interaction between their tandem DEDs and the C-terminus DED of FADD. However, the question of direct recruitment of cFLIP to the DISC (i.e., interaction with FADD_DED_) is rather controversial.^10,11^ Further recruitment of C8 enable these to dimerize and results in a significant conformational rearrangement in their catalytic caspase domains, which in turn leads to proximity-induced activation and initiation of a proteolytic apoptotic cascade.^12^

Several splice variants of cFLIP have been identified to date. At the protein level three isoforms have been described, the long cFLIP_L_ and the two short cFLIP_S_ and cFLIP_R_.^13,14^ All three isoforms contain the tandem DEDs (Figure 1a) which are highly homologous to the C8 tandem DEDs.^15^ cFLIP_L_ possesses a catalytically inactive caspase-like subdomain at its C-terminus while the two shorter splices lack this subdomain and are similar in architecture to the viral FLIP (vFLIP). Both cFLIP_S_ and cFLIP_R_ block the extrinsic apoptosis by preventing the C8 proximity activation at the DISC while the role of cFLIP_L_ in DR-induced apoptosis is more complicated. It has been demonstrated that based on the concentration of cFLIP_L_, it can act either as an antiapoptotic agent, i.e., diminishing the C8 activation at the DISC, or as a pro-apoptotic molecule enhancing C8 activation.^11^ However, there is much controversy in the literature on the mechanism of action of cFLIP. Some researchers believe that cFLIP compete directly with C8 for recruitment to the FADD binding site^16^ while others have proposed that there is no such direct competition; instead C8 and FLIP interact with different binding surfaces of FADD.^10^ Hughes et al. functionally reconstituted the DISC, and using quantitative LC-MS/MS and structure guided mutagenesis showed that not only is cFLIP binding to FADD noncompetitive but that cFLIP displays no or only a very weak interaction with FADD compared to C8.^11^ Instead, a cooperative C8 dependent process was described where FADD initially recruits C8, which in turn interacts and heterodimerizes with cFLIP via a hierarchical binding mechanism.

The aim of the present study is to identify the key hot spots in PPIs during the DISC formation and generate a reliable atomistic model of the multiprotein complex using computational protein docking techniques. However, despite remarkable improvements in docking algorithms and development of sophisticated sampling and scoring methods, it is still a difficult task to recognize and score the true positive complexes as top poses among the thousands of decoys generated.^17,18^ Furthermore, as the docking accuracy significantly depends on the quality of the target proteins used as input, it is difficult to estimate the accuracy for each resulting complex.^19^ In the current study, we have tried to overcome these shortcomings by introducing an exhaustive protein–protein docking meta-approach utilizing several available software to predict and explore pairwise protein–protein complexes which are subsequently merged into a full model of the hexagonal filament forming DISC structure.

To verify the computational results, we also performed a series of *in-cellulo* protein-fragment complementation assays (PCA) with the Renilla Luciferase enzyme as a reporter protein. As described elsewhere,^20^ the enzyme was separated into two fragments Nter and Cter, referred to as F1 and F2, and conjugated with the different cFLIP and C8 DED domains. This technique has long been used to rapidly confirm protein–protein or domain–domain interactions and allows through coexpression of fusion-proteins to easily get an idea of the relative interaction strength between two proteins. A long or more frequent interaction between the proteins enables a more optimal reconstitution of the luciferase, thereby giving a higher light emission. Based on this assay, a positive result is a good indication to further explore the protein–protein interaction in question, whereas a negative result (no, or few light emissions), can be attributed to conformational hindrance/lack of interaction between the two proteins. Identifying the hot spots in the DISC architecture and revealing the interacting surfaces is an essential step for designing new drugs with potential ability of modulating the PPIs in the DISC as either inhibitor or activator agents.

## Materials and Methods

### Homology Modeling

**Figure 2:**
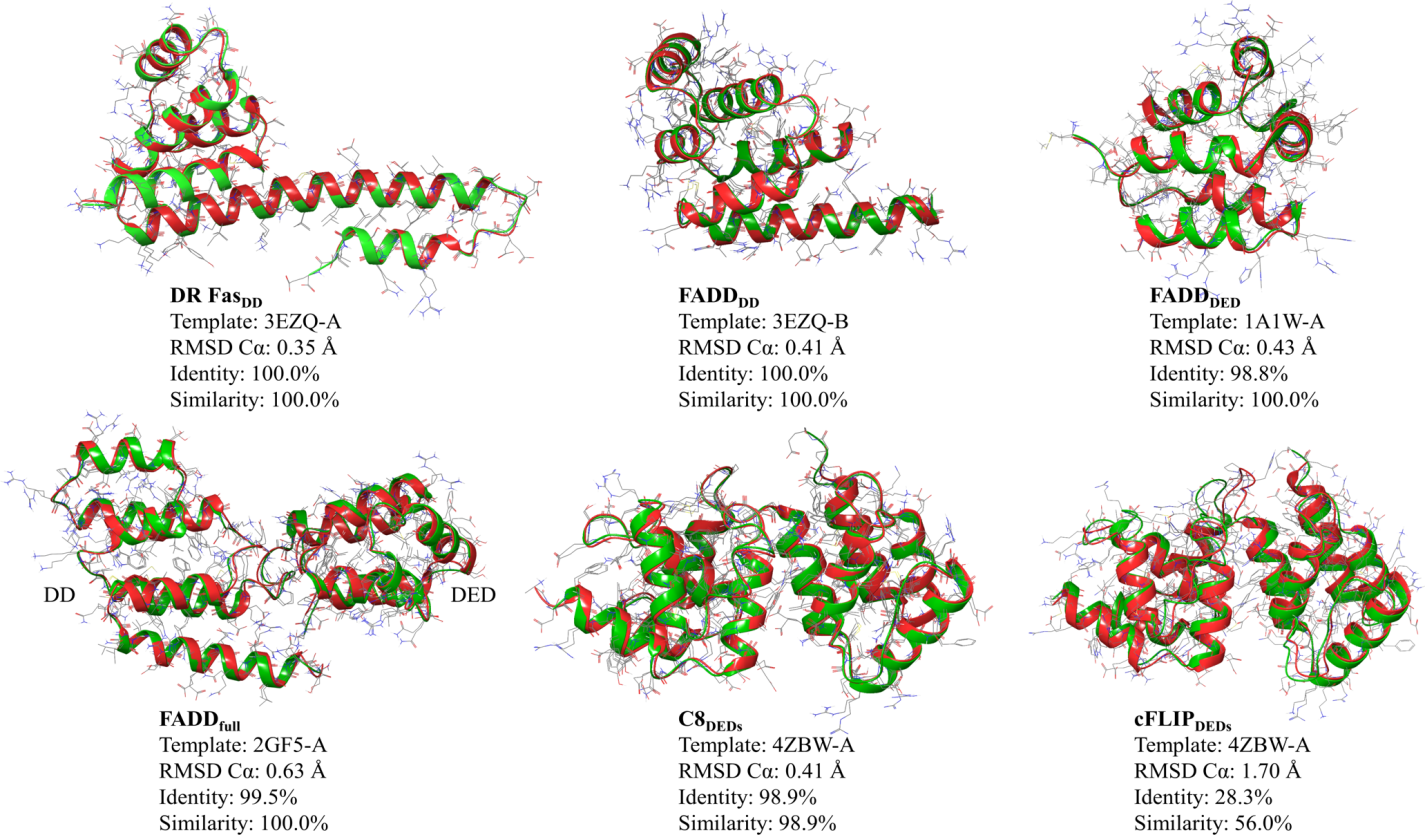
Superposed structures of each homology model on its template along with the RMSD Cα, sequence identity, and similarity percentage. The green and red ribbons present the templates and homology models for each set, respectively.

In order to ensure completeness of the protein structures, addition of missing loops, optimizing the orientation of side chains or, in the case of cFLIP, to generate a complete protein model, homology modeling of the different proteins was initially performed. All homology modeling was performed using default settings in YASARA version 19.9.17^21^ and the AMBER14 force field.^22^ Fas_DD_ (amino acids 223–335, UniProtKB: P25445), FADD_DD_ (93–191, UniProtKB: Q13158), FADD_DED_ (1–84, UniProtKB: Q13158), FADD_full_ (1–191, UniProtKB: Q13158), C8_DEDs_ (1–182, UniProtKB: Q14790), and cFLIP_DEDs_ (1–176, UniProtKB: O15519) were modeled using crystal structures with pdb-ids 3EZQ-A, 3EZQ-B, 1A1W-A, 2GF5-A, 4ZBW-A, and 4ZBW-A as templates, respectively. The Ramachandran plots of the homology models were generated using the molecular operating environment (MOE) software^23^ to assess their structural quality (Figures S1–S6). As the Ramachandran plots show, the majority of the residues in the Fas_DD_ (96.5%), FADD_DD_ (97.5%), FADD_DED_ (93.6%), FADD_full_ (95.5%), C8_DEDs_ (100.0%), and cFLIP_DEDs_ (97.1%) models were located in the core regions while the remaining residues were in the allowed regions with no outliers. Additional global and local quality estimation of the homology models were carried out using the Qualitative Model Energy Analysis (QMEAN) web server.^24^ The results of the quality assessments are presented in Figures S1–S6 and clearly confirm that the YASARA software has built very good quality homology models. The superposed structures of each homology model on its template along with the RMSD Cα, identity percent, and similarity percent are shown in Figure 2. Maestro Schrodinger 2020-2 was used for multiple sequence alignments and superpositions.

### Protein–Protein Docking

**Figure 3:**
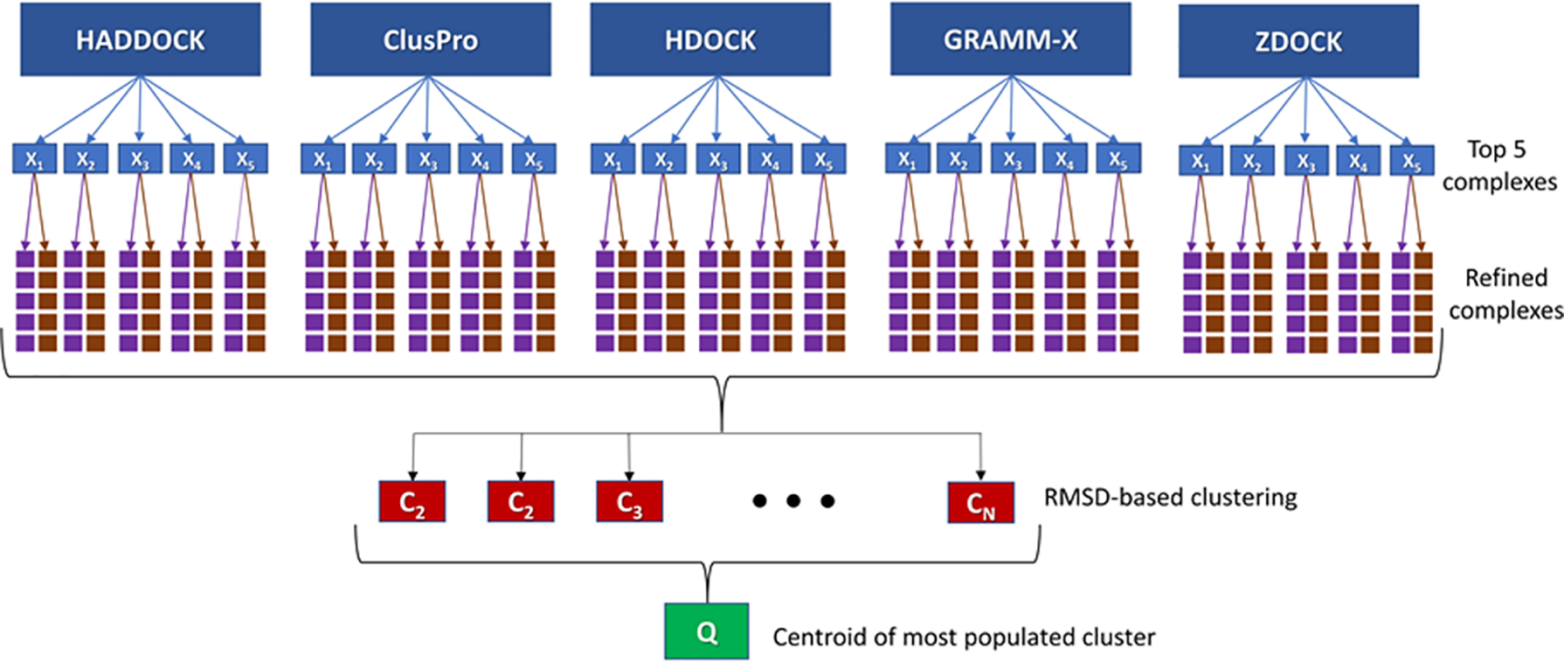
Flowchart of the strategy used in order to combine the benefits of five protein–protein docking engines, HADDOCK, ClusPro, HDOCK, GRAMM-X, and ZDOCK.

Many different tools and Web servers have been developed for protein–protein docking. The performance of each docking engine depends on the sampling algorithm, scoring function, and degree of flexibility (including complementary post processing). Moreover, the docking accuracy depends on the quality of the included protein structures, and thus it is not easy to estimate the accuracy for each individual case.^19^ We hence decided to use a new meta-strategy, combining the benefits of five of the most employed protein–protein docking engines (HADDOCK,^25,26^ ClusPro,^27,28^ HDOCK,^29,30^ GRAMM-X,^31^ and ZDOCK^32,33^), in order to obtain “consensus-based” predicted docking complexes. A summary of search algorithms and scoring functions implemented in each docking engine along with their reach-point URL is presented in Table S1. The flowchart presented in Figure 3 shows the approach used in this study. At the starting point, a series of nonblind protein–protein docking calculations (based on mutagenesis studies from the literature) were performed using the aforementioned docking engines, whereafter the top five predicted complexes (i.e., *X*_1_ to *X*_5_) from each docking engine were selected. The default setting and parameters were used in all docking engines. Each of the 25 docked poses was refined by means of the *GalaxyRefineComplex* tool using two different relaxation protocols.^34^ In the first protocol only distance restraints were applied, while the second protocol applied both distance and position restraints. The five lowest energy complexes from each refinement protocol were returned as the final 10 refined models for each initial complex. The thereby obtained 250 refined complexes were clustered based on the RMSD values of all heavy atoms using the “*clustering of conformer*” module implemented in Maestro Schrodinger (*i.e.*, *C*_1_–*C*_*N*_) (Schrödinger Release 2020-2: Maestro, Schrödinger, LLC, New York, NY, 2020.). The optimum number of clusters,*N*, was determined from Kelley penalty plots.^35^ Finally, the model nearest to the centroid of the most populated cluster was considered as the final docking pose, *Q*. To validate this new approach, we first successfully reproduced the crystal structures of the Fas_DD_–Fas_DD_ and Fas_DD_–FADD_DD_ protein–protein complexes (pdb-id: 3EZQ), as presented in the Results and Discussion.

### Molecular Dynamics Simulations

All molecular dynamics (MD) simulations were carried out for 300 ns in NPT ensembles using the Desmond MD simulator engine^36^ implemented in Schrödinger, with the OPLS3e force field.^37^ Water molecules were modeled using the TIP3P force field.^38^ Periodic boundary conditions were applied in all directions along with a 10 Å water buffer around the protein in a cubic simulation box. The net charge of the system was balanced using the proper number of counterions (i.e., Cl^–^/Na^+^), and the salt concentration was set to 150 mM to represent physiological conditions. Temperature (300 K) and pressure (1 atm) were controlled using the Nose–Hoover thermostat^39^ with the relaxation time of 1 ps and the Martyna–Tobias–Klein barostat^40^ with the relaxation time of 2 ps and isotropic coupling style, respectively. The nonbonded interactions were partitioned into short-range (van der Waals and electrostatic) and long-range (electrostatic) components. The short-range van der Waals and electrostatic interactions were modeled by 12–6 Lennard-Jones potential and Coulomb’s law within a cutoff radius of 10 Å, respectively. The long-range electrostatic forces were computed by the smooth particle mesh Ewald (PME) technique. The initial minimization and relaxation protocol consisted of (a) NVT Brownian dynamics with restraints on solute heavy atoms at *T* = 10 K for 100 ps, (b) NVT simulation at *T* = 10 K with restraints on solute heavy atoms for 12 ps, (c) NPT MD simulation at *T* = 10 K with restraints on solute heavy atoms for 12 ps, (d) NPT MD simulation at *T* = 300 K with restraints on solute heavy atoms for 12 ps, and (e) NPT MD simulation at *T* = 300 K without restraints for 24 ps. The minimization and relaxation step was followed by a 300 ns production step in each system.

### Protein-Fragment Complementation Assay (PCA)

All cDNA plasmids which encode the Renilla Luciferase fusion proteins were provided by our collaborators of the INSERM Unit U1242, Centre Eugene Marquis, Rennes, France (see Supplementary Figure S15, for additional information). HEK293T cells were cultured in DMEM supplemented with 10% heat-inactivated FCS (v/v) and 2 mM l-glutamine at 37 °C in a 5% CO_2_ incubator. The cells were plated 1 day prior transfection onto 35 mm dishes. The standard calcium phosphate transfection protocol was followed.^41^ A 1:1 DNA ratio was used for each cotransfection. As described by Stefan et al.,^20^ after 24 h of transfection, cells were harvested, washed with PBS, and resuspended in FBS free Opti-MEM medium. Cells (∼10^6^) were incubated with 5 μM of Coelenterazine-h (Promega), and the luminescence was assessed using a POLARstar Omega luminescent plate reader (BMG Labtech). In all PCA analyses, the control consisted of the coexpression of the plasmid encoding the N- and C-term fragments of the luciferase to estimate the enzyme self-assembly.

### Data and Software Availability

Amino acid sequences of all proteins listed above were retrieved from Uniprot: https://www.uniprot.org/. All protein crystal structures were downloaded from the Protein Data Bank, https://www.rcsb.org/. Homology modeling was performed using YASARA, available at http://yasara.org/ (maintenance fee based) using default settings. The quality of the obtained protein models were assessed using Ramachandran plots in MOE, www.chemcomp.com (paid license), and through the Swiss-Model QMEAN server: https://swissmodel.expasy.org/qmean/.

The protein–protein docking was performed using the free Web servers HADDOCK https://alcazar.science.uu.nl/services/HADDOCK2.2/, ClusPro https://cluspro.org/home.php, HDOCK http://hdock.phys.hust.edu.cn/, GRAMM-X http://vakser.compbio.ku.edu/resources/gramm/grammx, and ZDOCK http://zdock.umassmed.edu/, using default settings unless indicated in text. Complex refinements were performed using the web server GalaxyWEB http://galaxy.seoklab.org/cgi-bin/submit.cgi?type=COMPLEX.

Schrodinger 2020-2 (www.schrodinger.com; paid license) was used for complex clustering (clustering of conformer module), superposition of structures (Maestro), and MD simulations (Desmond), MM-GBSA energies, and alanine scanning (BioLuminate) using the settings as described above.

Data sets with complex structures and MD trajectories are available freely via the Zenodo repository, as 10.5281/zenodo.4064682.

## Results and Discussion

### Fas_DD_–Fas_DD_ Complex

**Figure 4:**
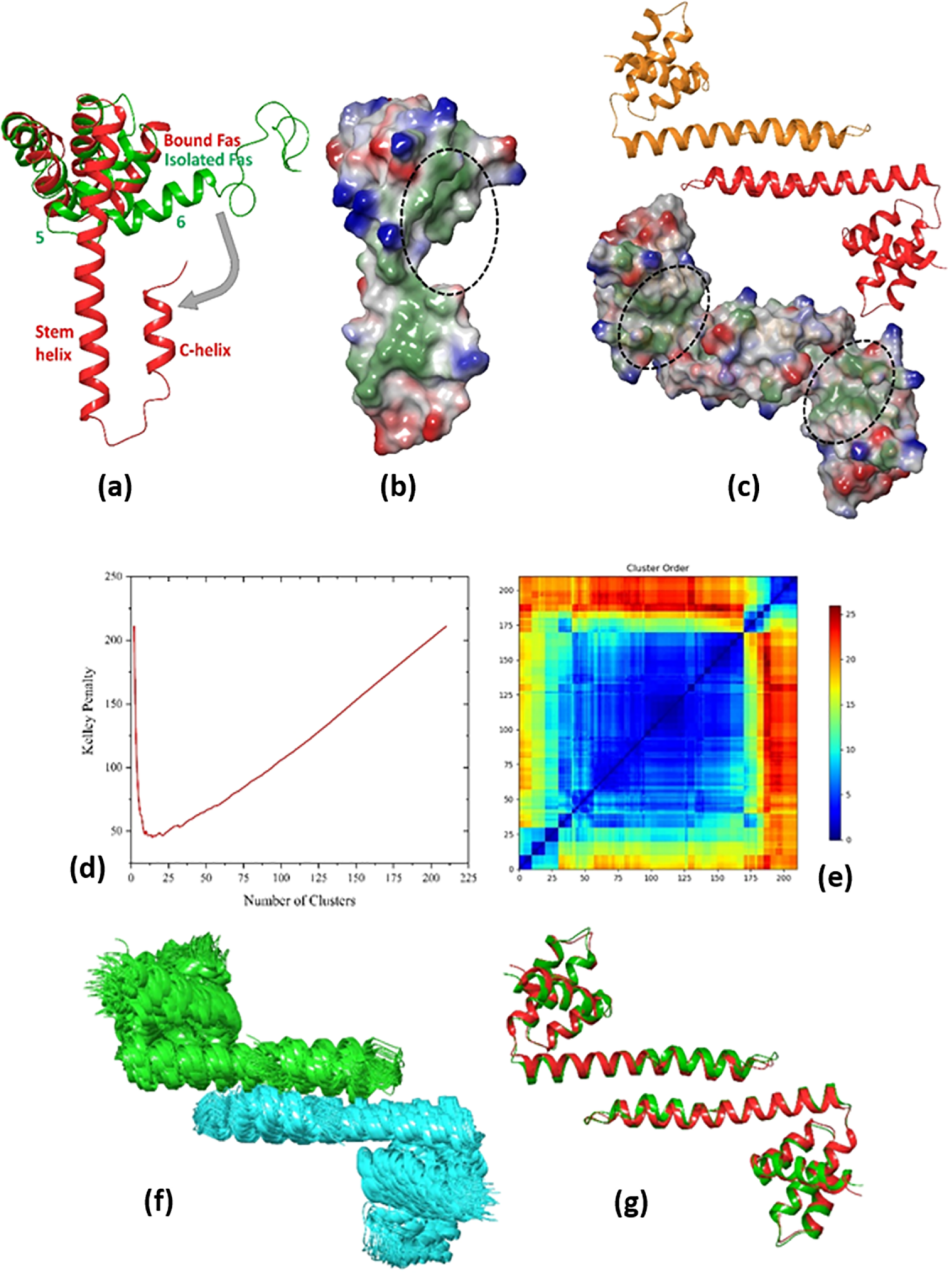
(a) Conformational rearrangement from closed isolated Fas_DD_ (pdb-id: 1DDF, green) to open bound Fas_DD_ (pdb-id: 3EZQ, red). (b) Fas_DD_ opening discloses a hydrophobic patch that is the binding site of FADD_DD_, shown with dashed circles. (c) Fas_DD_ rearrangement allows for homodimerization of two open Fas molecules through interactions between their stem helices in a Fas–Fas bridge conformation. (d) Kelley penalty plot and (e) distance matrix from Fas_DD_–Fas_DD_ clustering. (f) Most populated cluster with 83 members. Fas_1,DD_ and Fas_2,DD_ are presented in green and cyan colors, respectively. (g) Predicted complex (red) superposed on the crystal structure (green) (pdb-id: 3EZQ) with a Cα RMSD value of 1.1 Å.

It has been demonstrated that during the DL stimulation, the extracellular domains of Fas DR form homotrimeric complexes.^42^ The extracellular aggregation of Fas subsequently induces trimerization of the cytosolic globular units of Fas_DD_ (residues 230–285) through proline motif-mediated homoagglomeration of its transmembrane helices.^43^ Scott et al.^44^ showed that compared with the isolated solution structure of the Fas_DD_,^45^ Fas_DD_ in the trimeric complex undergoes a significant conformational rearrangement. During this rearrangement (referred to as “opening”), helix 6 (residues C304–T319) shifts and fuses with helix 5 (residues K287–L303) to form a long stem helix and simultaneously a new short “C-helix” (residues N326–L336) forms at the C-terminus of Fas (Figure 4a). The Fas opening has two consequences: first it discloses a hydrophobic patch which serves as the binding site of FADD_DD_ (Figure 4b and c), and second it allows homodimerization of two open Fas molecules through interactions between their stem helices in a Fas–Fas bridge conformation (Figure 4c).^44^

The Fas–Fas bridge dimer is a minimal requirement for a stable Fas–FADD complex.^46^ However, it has been hypothesized that an extensive network is formed by dimerization of Fas_DD_ stem helices located in adjacent trimeric Fas_DDs_ interacting through the globular part of the DD^9^ as shown in Figure 1b and that these will eventually form the basis of procaspase-8 filament formation. Hence, as the starting point, the Fas–Fas bridge complex was rebuilt using the strategy outlined earlier. Based on mutagenesis studies, the two residues K299 and I310 located in the stem helix have been determined to be involved in the Fas–Fas bridge formation.^44^ These were therefore defined as interacting residues (Table S2) in the nonblind protein–protein docking calculations. The results of the conformation clustering are presented in Figure 4d–g. As Figure 4d indicates, the optimum number of clusters from the Kelley penalty plot is 14, and the associated distance matrix is presented in Figure 4e. The most populated cluster with 83 members is shown in Figure 4f. The standard deviation, population, and average RMSD from the centroid of each cluster along with the relative RMSD values of the centroid of each cluster compared to the centroid of the most populated one are presented in Figure S7a. Figure 4g shows the predicted complex superposed on the crystal structure (pdb-id: 3EZQ) with a Cα RMSD value of 1.1 Å. It should be mentioned that the total number of conformers in this case was 210 instead of 250 since the GRAMM-X server predicted only one pose.

**Scheme 1:**
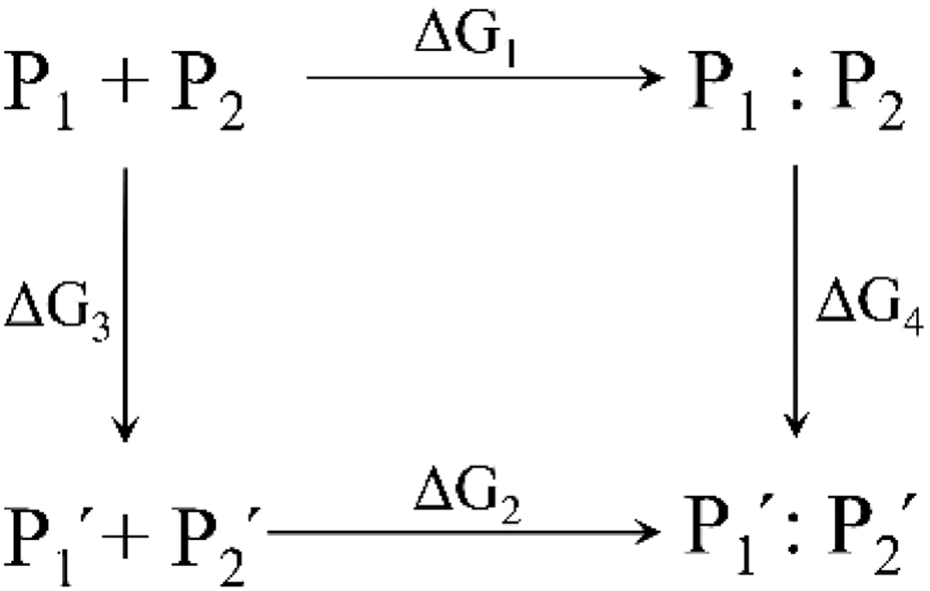
Thermodynamic Cycle Used in Alanine Scanning Calculation to Estimate the Change in Protein Binding Affinity Due to Residue Mutation

The Schrödinger package was employed to calculate the free energy of binding between the two Fas_DD_ molecules in the predicted complex using the molecular mechanics generalized Born surface area (MM-GBSA) technique,^47^ giving the value of −116.7 kcal mol^–1^. Moreover, the most important interacting residues (hot spots) engaging in Fas_DD_–Fas_DD_ complex formation were determined based on the change in protein binding affinity (ΔAff) upon residue mutation to alanine, using BioLuminate alanine scanning calculations^48^ as implemented in the Schrodinger package. The change in binding affinity is calculated from a thermodynamic cycle as presented in Scheme 1.^48^

In Scheme 1, P_1_ and P_2_ are the two initial proteins and P_1_′ and P_2_′ are the corresponding mutated ones. P_1_ + P_2_ and P_1_′ + P_2_′ represent the separated proteins whereas P_1_:P_2_ and P_1_′:P_2_′ show the resulting protein complexes. The change in protein binding affinity can be calculated as follows:
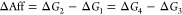
1Whereas Δ*G*_1_ and Δ*G*_2_ can be measured experimentally, Δ*G*_3_ and Δ*G*_4_ are calculated which may benefit from cancellation of errors in the computational models. The free energy calculations were done with Prime MM-GBSA which uses an implicit solvation model. A positive value indicates that the muted proteins bind worse than the parent ones. The results of the alanine scanning calculations are presented in Table 1. As this table indicates, residues R328, L303, V335, K299, and F327 have the highest contribution to the Fas_DD_–Fas_DD_ stem helix binding affinity (Figure S8).

**Table 1:** Residues with the Highest Contribution to the Fas_DD_–Fas_DD_ Binding Affinity Identified by Alanine Scanning Calculations^a^

mutation	ΔAff (Fas1) kcal mol^–1^	ΔAff (Fas2) kcal mol^–1^	average ΔAff kcal mol^–1^
R328A	16.3	9.6	13.0
L303A	11.9	13.5	12.7
V335A	11.4	14.0	12.7
K299A	12.9	12.0	12.5
F327A	16.3	8.4	12.4
K300A	10.9	10.8	10.9
I314A	5.5	15.0	10.3
I318A	8.6	8.6	8.6
I331A	6.8	5.5	6.2
I310A	5.2	6.5	5.9

a Only mutated residues with ΔAff > 5 kcal mol^–1^ are listed.

### Fas_DD_–FADD_DD_ Complex

**Figure 5:**
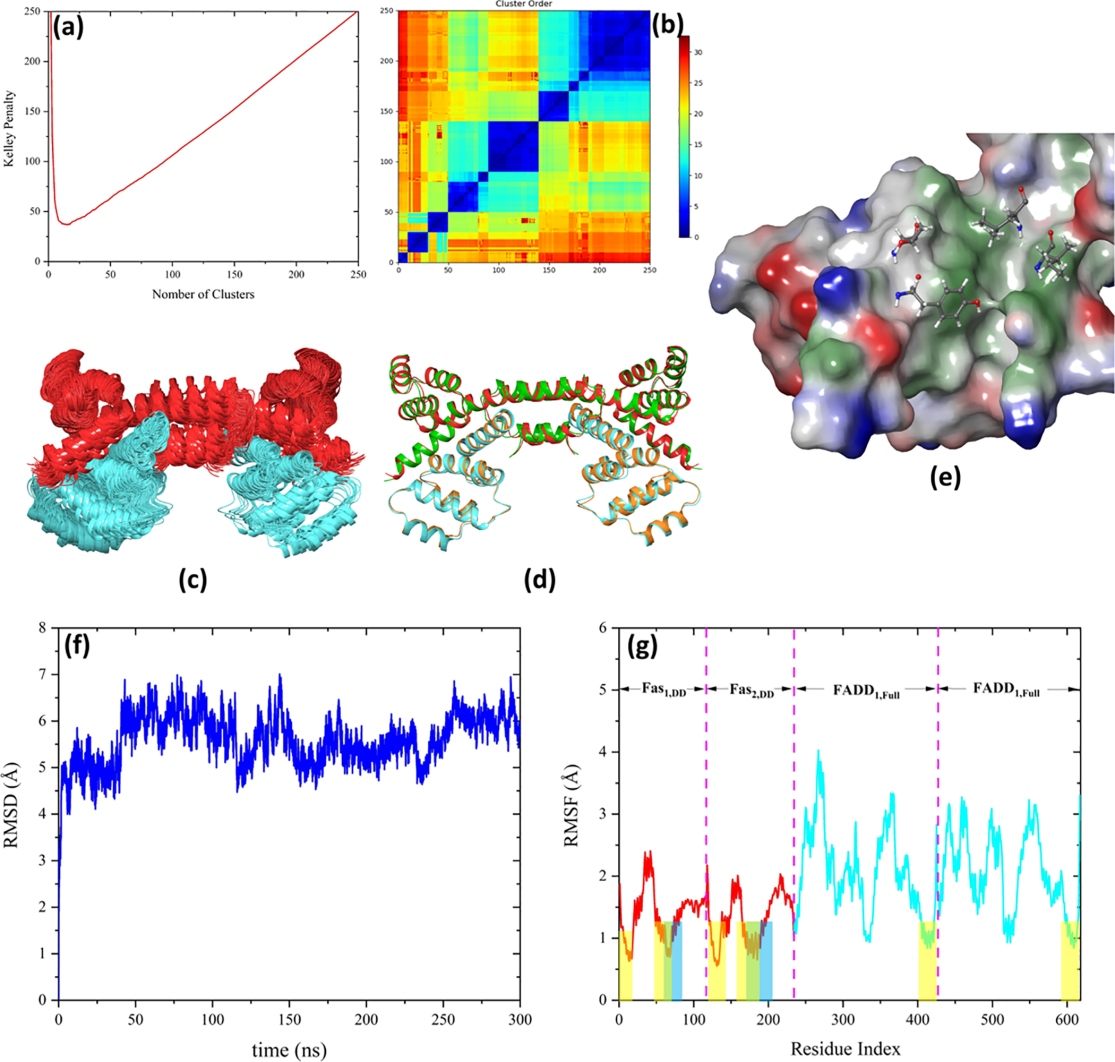
(a) Kelley penalty plot and (b) distance matrix from the Fas_DD_–FADD_DD_ docking. (c) Most populated tetrameric clusters with 61 and 50 members corresponding to the Fas_1_ DD–FADD_1_ DD and Fas_2_ DD–FADD_2_ DD binding, respectively. The Fas_DDs_ and FADD_DDs_ are presented in red and cyan, respectively. (d) Predicted complex (green: Fas_DDs_, cyan: FADD_DDs_) superposed on the crystal structure (pdb-id: 3EZQ) (red: Fas_DDs_, orange: FADD_DDs_) with a Cα RMSD value of 1.9 Å. (e) Y232, T235, I295, and L298 are the central residues in the Fas_DD_ hydrophobic patch presented in ball–stick representation. (f) Cα RMSD and (g) RMSF plots of the tetrameric Fas_DD_–FADD_DD_ complex during 300 ns MD simulation. The residues corresponding to each molecule engaged in the complex formation are shown in the RMSF panel. The blue and yellow dashed areas in the RMSF plot show the interacting regions for Fas_DD_–Fas_DD_ (blue) and Fas_DD_–FADD_DD_ (yellow) interactions, respectively.

One of the consequences of Fas opening is the disclosure of a hydrophobic patch that will be the binding site of FADD_DD_ (Figure 4b and c). Protein surface analyses identified a large hydrophobic patch with a surface area of 735 Å^3^ and a scoring value of 425 kcal mol^–1^, out of which the Fas_DD_–FADD_DD_ binding interface constitutes a large part. According to the surface analysis, the central residues of Fas in the Fas_DD_–FADD_DD_ binding interface are Y232, T235, I295, and L298 (Figure 5e); these residues along with hydrophobic residues of FADD_DD_ i.e., L172, L176, and L186,^44^ were considered as interacting residues in the protein–protein docking (Table S2). The results of the conformational clustering are presented in Figure 5. The Kelley penalty plot indicated that the optimum number of clusters is 15 (Figure 5a), and the associated distance matrix is presented in Figure 5b. The standard deviation, population, and average RMSD from the centroid of each cluster along with the relative RMSD values of the centroid of each cluster to the centroid of the most populated one are presented in Figure S7b. The two most populated clusters, with 60 and 50 members, respectively correspond to the Fas_1,DD_–FADD_1,DD_ and Fas_2,DD_–FADD_2,DD_ interactions (Figures 5c and S7b). Figure 5d presents the predicted complex superimposed on the crystal structure (pdb-id: 3EZQ), giving a Cα RMSD value of 1.9 Å.

The free energy of binding of Fas_DD_–FADD_DD_ was determined to be −155.1 kcal mol^–1^ by means of MM-GBSA calculations. The hot spots in the Fas_DD_–FADD_DD_ interaction were identified using alanine scanning calculations and are presented in Table 2 and Figure S9. The data is in good agreement with that from the protein surface analysis. The central residues identified in the hydrophobic patch of the open Fas_DD_ by protein surface analysis i.e., Y232, T235, I295, and L298 (Figure 5e) and FADD_DD_ i.e., L176, and L186, were also identified among the hot spot residues in the Fas_DD_–FADD_DD_ interacting region in the alanine scanning calculations. In order to check the stability of the tetrameric Fas–FADD complex, the DD of two FADD_full_s were superposed on the DD of FADD in the Fas_DD_–FADD_DD_ complex and the tetramer was subjected to 300 ns MD simulation. The resulting RMSD and RMSF plots are shown in Figure 5. As Figure 5 indicates, the complex was highly stable after the initial 50 ns of the MD simulation and the fluctuations in the two FADDs were larger than those of the Fas_DDs_.

**Table 2:** Residues with the Highest Contribution to the Fas_DD_–FADD_DD_ Binding Affinity Identified by Alanine Scanning Calculations^a^

mutation in Fas	ΔAff kcal mol^–1^	mutation in FADD	ΔAff kcal mol^–1^
**S225A**	12.2	R189A	19.2
K288A	11.6	L186A	16.2
T235A	10.5	L176A	14.6
Y232A	8.7	R142A	12.6
L224A	8.6	V180A	9.2
N302A	8.3	Q182A	8.2
I295A	8.3	N102A	7.3
R328A	7.9	N107A	7.2
L298A	6.6	Q187A	7.2
		N136A	6.4
		T138A	6.3

a Only mutated residues with ΔAff > 5 kcal mol^–1^ are listed.

Using 150 ns MD simulations, Yan et al.^46^ demonstrated that FADD binding to Fas stabilize the overall structure of the complex and resulted in a reduced degree of anticorrelated as well as correlated motion of the residues in FADD. They concluded that dynamical motion of FADD residues causes the relative conformational changes between FADD_DED_ and FADD_DD_, leading to exposure of the α1 and α4 helices of the FADD_DED_ making them available to recruit C8 into the DISC. However, clustering analysis of our MD simulation trajectory in the last 150 ns (repeated three times with different initial atomic velocity distributions) showed that albeit conformational changes occur in FADD, there is not enough room around the α1/α4 helices of FADD_DED_ in the complex (Figure S10). According to the current results, the FADD_DED_ surface formed by the α2/α5 helices may instead be the binding site for C8 and cFLIP molecules. This result is in agreement with the findings of Majkut et al.^10^ and Hughes et al.,^11^ where they showed that C8 binds to the α2/α5 surface of FADD_DED_ instead of α1/α4. This implies that only one C8 or cFLIP molecule at a time can bind directly to the FADD_DED_. However, additional direct C8–C8 and C8–cFLIP interactions would change the 1:1 stoichiometric ratio.^49^ Indeed, some studies have shown that each FADD molecule can recruit between 6 and 10 DED-containing proteins.^49,50^ Based on the protein–protein docking results and previous experimental studies, the mechanism of the Fas_DD_–Fas_DD_ bridge and Fas_DD_–FADD_full_ formation as illustrated in Figure 1b is hence elucidated.

### FADD_DED_–C8_DEDs_/cFLIP_DEDs_ Complexes

**Figure 6:**
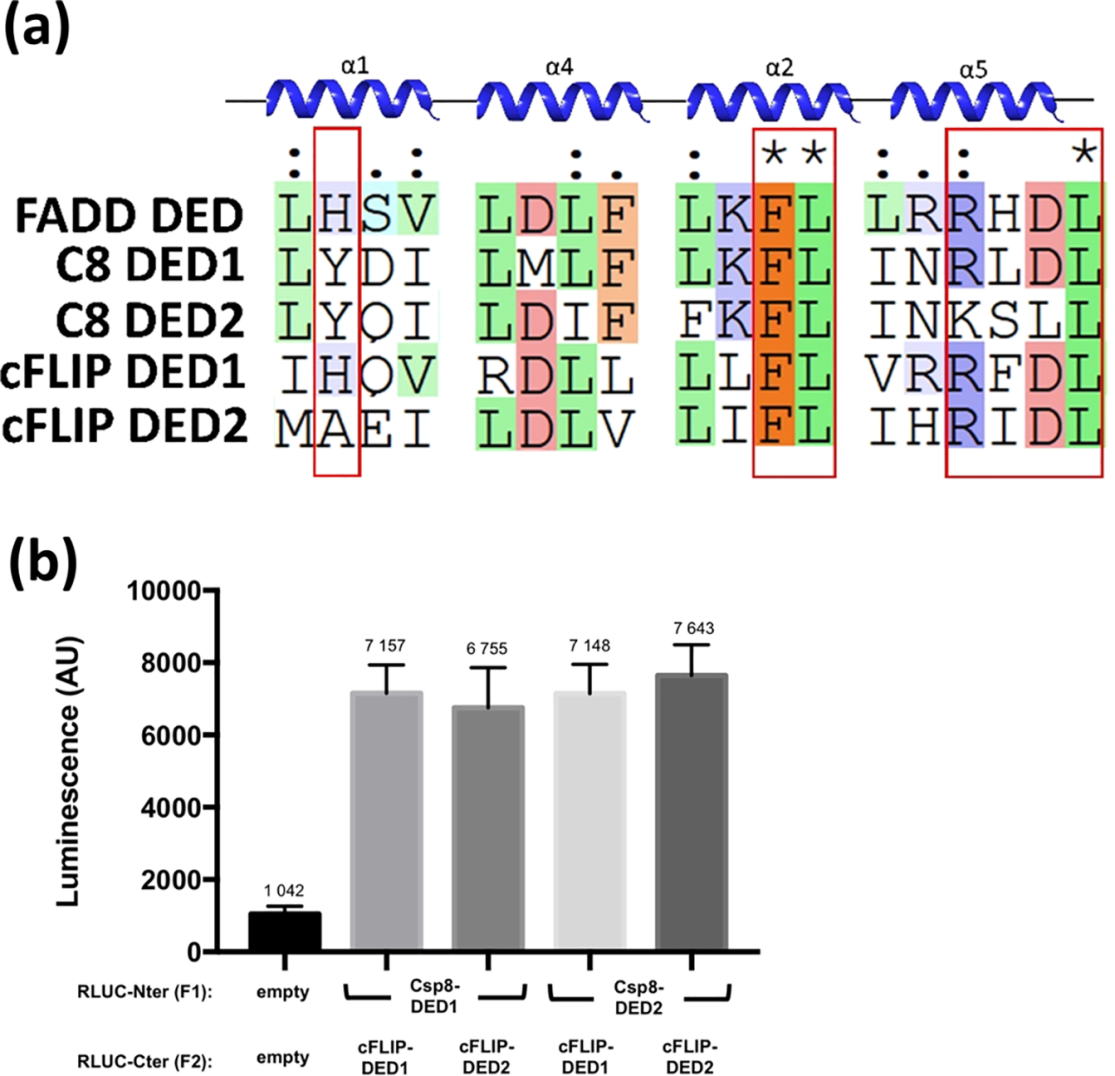
(a) Multiple sequence alignment of residues that form part of the hydrophobic patches in the α1/α4 and α2/α5 surfaces of FADD_DED_, C8_DEDs_, and cFLIP_DEDs_. H/Y residues in α1, the FL motif in α2, and the RxDL motif in α5 are highlighted. Maestro Schrodinger 2020-2 was used for the alignment. (b) PCA results for intersubdomain interactions of C8_DED1_ and C8_DED2_ with cFLIP_DED1_ and cFLIP_DED2_. C8_DEDs_ and cFLIP_DEDs_ domains were coexpressed in HEK293T as indicated. Negative control is the result of coexpression of empty vectors (containing only a subunit of the luciferase as indicated). The luminescence is expressed in arbitrary unit (AU). Data represent mean ± SD of three independent experiments for each cotransfection.

The next step in the DISC formation is the recruitment of C8 and/or cFLIP through homotypic DED interactions with FADD molecules. Two hydrophobic surfaces have been identified for DED-containing proteins i.e. α1/α4 and α2/α5.^10^Figure 6 shows the multiple sequence alignment of residues that form part of the hydrophobic patches in the α1/α4 and α2/α5 surfaces, which are highly conserved in DED-containing proteins.^10^ The FL motif, located in the α2 helix (residues S18 to C27 in FADD_DED_), belongs to the hydrophobic patch of almost all DED-containing proteins including FADD_DED_, C8_DED1_, C8_DED2_, cFLIP_DED1_, and cFLIP_DED2_ (residues F25–L26, F24–L25, F122–L123, F23–L24, and F114–L115, respectively). The hydrophobic nature of H9 in the α1 helix of FADD_DED_ (residues F4–S14) is also conserved, however, as Y8 in C8_DED1_, Y10 in C8_DED2_, H7 in cFLIP_DED1_, and A98 incFLIP_DED2_. Moreover, the RxDL motif located in the α5 helix of DED-containing proteins (residues T60–R71 in FADD_DED_) is also highly conserved.^51^ It has been assumed that the intermolecular interactions between FADD_DED_ and C8_DEDs_/cFLIP_DEDs_ follow the same principle as the C8_DED1_– C8_DED2_ and cFLIP_DED1_– cFLIP_DED2_ intramolecular interactions in which the FL motifs in the α2 helix of one DED bind into the hydrophobic pocket in the groove between α1 and α4 of the next DED. The PCA results clearly support this assumption since each C8_DED1_ and C8_DED2_ domain could interact with each cFLIP_DED1_ or cFLIP_DED2_, without any preference (Figure 6b). This hypothesis has also been confirmed in mutagenesis experiments.^10,11^ We thereby defined these as interacting residues in the protein–protein docking procedure (Table S2).

**Figure 7:**
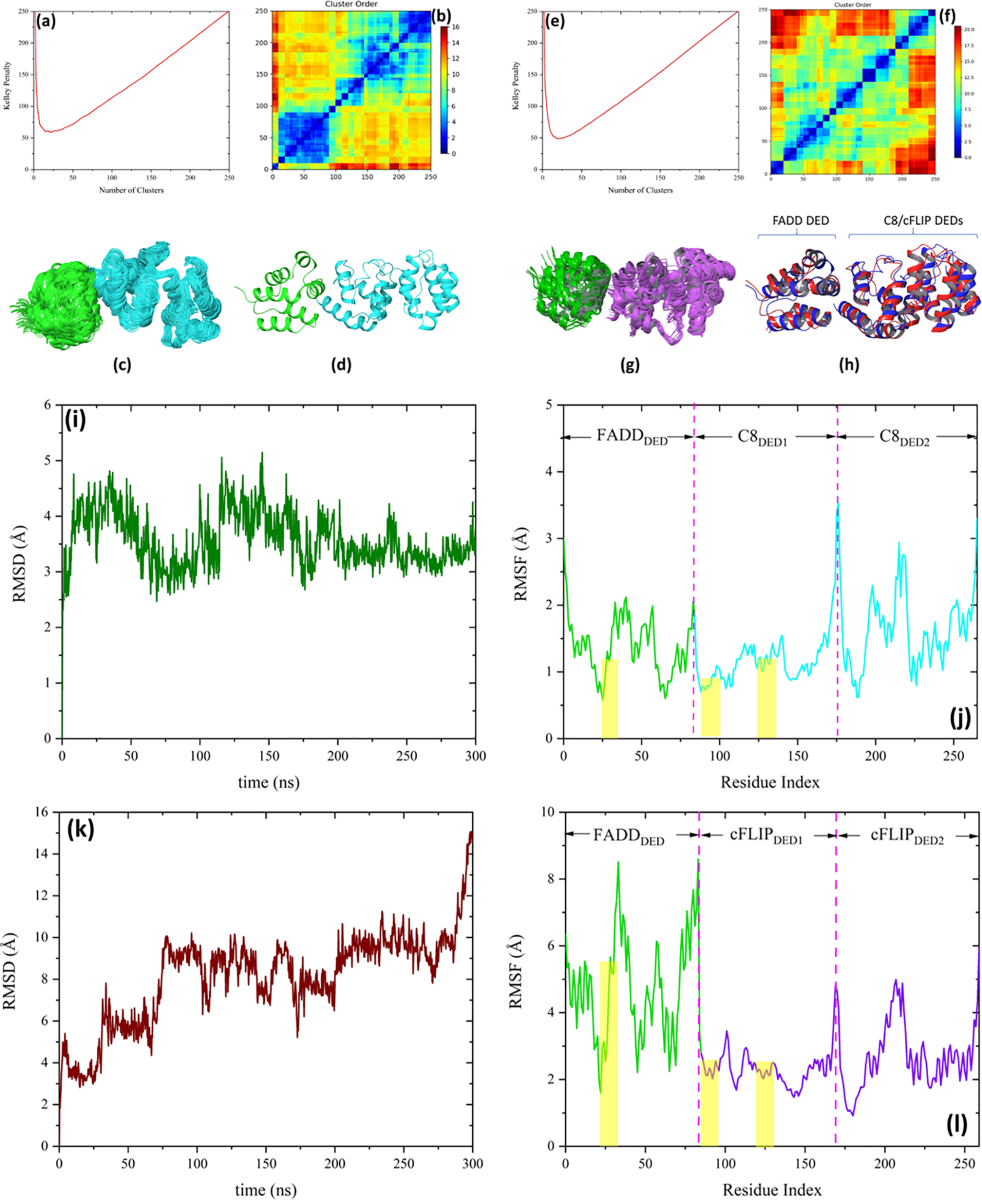
(a) Kelley penalty plot and (b) distance matrix from FADD_DED_–C8_DEDs_ docking. (c) Most populated cluster with 70 members and (d) the predicted complex based on the most populated cluster. FADD_DED_ and C8_DEDs_ are presented in green and cyan colors, respectively. (e) Kelley penalty plot and (f) distance matrix from FADD_DED_–cFLIP_DEDs_ docking. (g) Most populated cluster with 30 members. FADD_DED_ and cFLIP_DEDs_ are presented in green and purple colors, respectively. (h) Predicted complex of FADD_DED_–cFLIP_DEDs_ (red) superposed on the predicted complex of FADD_DED_–C8_DEDs_ (blue) with the Cα RMSD value of 4.5 Å. (i) RMSD and (j) RMSF plots of FADD_DED_–C8_DEDs_ and (k) RMSD and (l) RMSF plots FADD_DED_–cFLIP_DEDs_, during 300 ns MD simulation. The residues corresponding to each molecule engaged in the complex formation are shown in the RMSF panels. The yellow dashed areas in the RMSF plots show the interacting regions.

The results of the conformation clustering and protein–protein docking of FADD_DED_–C8_DEDs_ and FADD_DED_–cFLIP_DEDs_ are presented in Figure 7. The Kelley penalty graphs (Figure 7a and e) indicate that the optimum number of clusters are 15 and 18 for FADD_DED_–C8_DEDs_ and FADD_DED_–cFLIP_DEDs_, respectively. The associated distance matrixes are presented in Figure 7b and f, respectively. For FADD_DED_–C8_DEDs_, the conformational clustering led to a cluster populated with 70 members (Figure 7c), while for FADD_DED_–cFLIP_DEDs_, the 250 conformers almost evenly populated the 18 clusters with the most populated one containing 30 members (Figure 7g). The standard deviation, population, and average RMSD from the centroid of each cluster along with the relative RMSD values of the centroid of each cluster to the centroid of the most populated one, for FADD_DED_–C8_DEDs_ and FADD_DED_–cFLIP_DEDs_ interactions, are presented in Figure S7c and d, respectively.

The predicted complexes of FADD_DED_–C8_DEDs_ and FADD_DED_–cFLIP_DEDs_ are presented in Figure 7d and h (the latter superposed on the FADD_DED_–C8_DEDs_ complex with a RMSD Cα value of 4.5 Å), respectively. The MM-GBSA calculations indicated that the free energy of binding of the FADD_DED_–C8_DEDs_ complex (−60.9 kcal mol^–1^) is considerably stronger than that of FADD_DED_–cFLIP_DEDs_ (−37.0 kcal mol^–1^). The key hot spot residues engaging in FADD_DED_–C8_DEDs_ and FADD_DED_–cFLIP_DEDs_ interactions were determined using alanine scanning calculations and are presented in Tables 3 and 4 and Figure S11. As Table 3 and Figure S11 show, the FL motif of FADD_DED_, residues F25 and L26 (Figure 6a), and the conserved hydrophobic residue Y8 in the α1/α4 region of C8_DED1_ (Figure 6a) were identified by alanine scanning among the residues with largest contribution to the FADD_DED_–C8_DEDs_ binding affinity. However, as Table 4 indicates, residues in FADD_DED_–cFLIP_DEDs_ interface are less involved in PPIs confirming its lower free energy of binding value compared to FADD_DED_–C8_DEDs_.

**Table 3:** Residues with the Largest Contribution to the FADD_DED_–C8_DEDs_ Binding Affinity Identified by Alanine Scanning Calculations^a^

mutation in FADD	ΔAff kcal mol^–1^	mutation in C8	ΔAff kcal mol^–1^
F25A	17.4	R5A	20.1
R71A	14.1	Y8A	18.1
L26A	9.7	Q49	12.7
		M1A	8.6
		S4A	8.0
		E50A	7.2

a Only mutated residues with ΔAff > 5 kcal mol^–1^ are listed.

**Table 4:** Residues with the Largest Contribution to the FADD_DED_–cFLIP_DEDs_ Binding Affinity Identified by Alanine Scanning Calculations^a^

mutation in FADD	ΔAff kcal mol^–1^	mutation in C8	ΔAff kcal mol^–1^
R72A	14.4	E10A	11.7
E22A	11.0	R38A	5.8
R71A	10.5		
T21A	5.4		
F25A	5.1		

a Only mutated residues with ΔAff > 5 kcal mol^–1^ are listed.

Here, 300 ns MD simulations were conducted in order to validate the stabilities of the complexes predicted from the protein–protein docking calculations. Figures 7i–l shows the Cα RMSD and RMSF plots of the FADD_DED_–C8_DEDs_ and FADD_DED_–cFLIP_DEDs_ complexes during the 300 ns MD simulations. As seen, the interaction between FADD_DED_ and C8_DEDs_ is sufficiently strong (confirming the MM-GBSA calculation) to stabilize the protein complex during the MD simulation. The FADD_DED_–cFLIP_DEDs_ complex, on the other hand, was not stable and underwent significant structural reorientation at the binding surface whereafter the two molecules separated. In other to deeply evaluate the binding profile of FADD_DED_-C8_DEDs_ and FADD_DED_–cFLIP_DEDs_ complexes, we examined how the native residue contacts (from the docking poses) were maintained throughout the MD simulation trajectory. The native residue contacts were specified by any atomic interactions, within a cutoff radius of 5 Å, between the residues with the highest contribution to the binding affinity (>10 kcal mol^–1^ from Tables 3 and 4) in one protein (i.e., FADD_DED_) and all other residues of the other protein (i.e., C8_DEDs_ and cFLIP_DEDs_) and *vice versa*. The results are presented in Figure S12a and b. While almost all the native contacts in the FADD_DED_–C8_DEDs_ complex were maintained during 300 ns MD simulation, the corresponding native contacts were diminished and disappeared in the FADD_DED_–cFLIP_DEDs_ complex. The only native residue contact which was maintained in the FADD_DED_–cFLIP_DEDs_ complex is the interaction between residues E22 and R45 in FADD_DED_ and cFLIP_DEDs_, respectively. Figure S12c and d shows the first (*t* = 0 ns) and last (*t* = 300 ns) snapshots of the MD trajectory for FADD_DED_–C8_DEDs_ and FADD_DED_–cFLIP_DEDs_ complexes, respectively. These results support the view that cFLIP recruitment to the DISC is a hierarchical and cooperative process where FADD initially recruits C8 which in turn may recruit and heterodimerize with cFLIP. The results of the protein–protein docking and MD simulations are in good agreement with the experimental data reported by Hughes et al.^11^ Majkut et al.^10^ also found that C8 displays stronger affinity to the α2/α5 surface of FADD_DED_ than what cFLIP does. Moreover, the results from the experimental study by Fu et al. supports the weak interaction between FADD and cFLIP observed herein.^52^

### C8_DEDs_–C8_DEDs_/cFLIP_DEDs_ Complexes

**Figure 8:**
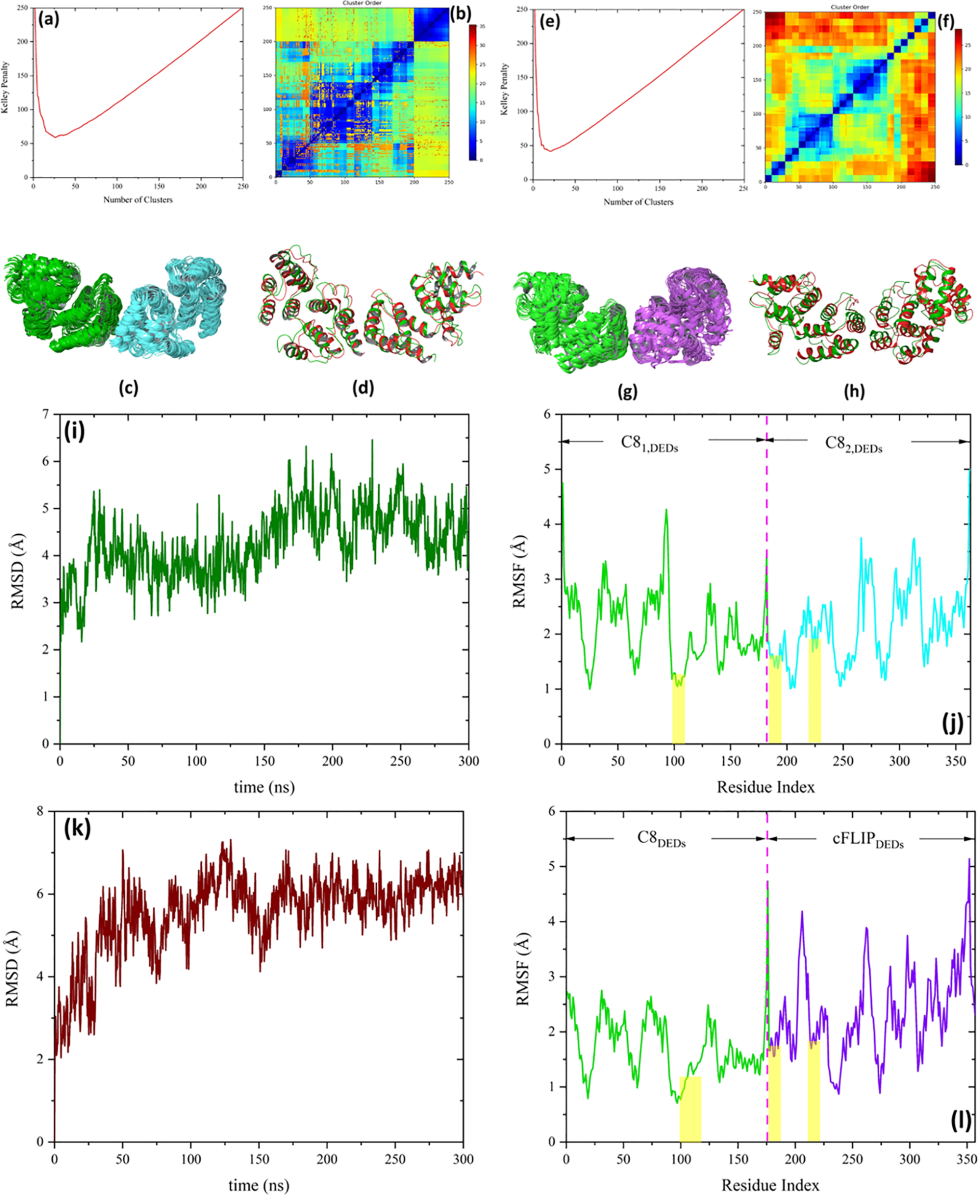
(a) Kelley penalty plot and (b) distance matrix from the C8_DEDs_–C8_DEDs_ docking. (c) Most populated cluster with 57 members. C8_1,DEDs_ and C8_2,DEDs_ are presented in green and cyan colors, respectively. (d) Predicted C8_DEDs_ homodimeric complex (red) superposed on the Cryo-EM structure of the C8_DEDs_ filament (green) (pdb-id: 5L08) with Cα RMSD value of 3.0 Å. (e) Kelley penalty graph and (f) distance matrix from the C8_DEDs_–cFLIP_DEDs_ docking. (g) Most populated cluster with 40 members. C8_DEDs_ and cFLIP_DEDs_ are presented in green and purple colors, respectively. (h) Predicted C8_DEDs_–cFLIP_DEDs_ heterodimeric complex (red) superposed on the crystallographic structure of the C8_DEDs_ filament (green) (pdb-id: 5L08) with Cα RMSD value of 3.4 Å. (i and k) Cα RMSD and (j and l) RMSF plots of C8_DEDs_–C8_DEDs_ and C8_DEDs_–cFLIP_DEDs_, respectively, during 300 ns MD simulations. The residues corresponding to each molecule engaged in complex formation are shown in the RMSF panels. The yellow dashed areas in the RMSF plots show the interacting regions.

Similar to the FADD_DED_–C8_DEDs_/cFLIP_DEDs_ case, we defined the conserved residues in the FL motif of C8_DED1_ and the α1/α4 hydrophobic pocket of C8_DED2_/cFLIP_DED2_ as interacting residues in the protein–protein docking procedure (Table S2). The Kelley penalty plot in Figure 8a shows that the optimum number of clusters for C8_DEDs_–C8_DEDs_ clustering is 24, and the associated distance matrix is presented in Figure 8b. The most populated cluster with 57 members is illustrated in Figure 8c. The standard deviation, population, and average RMSD from the centroid of each cluster along with the relative RMSD values of the centroid of each cluster and the centroid of the most populated one (as reference) are presented in Figure S7e. The predicted complex was in good agreement with the cryo-EM crystallographic structure of C8_DEDs_ filament assembly (pdb-id: 5L08) reported by Fu et al.^52^Figure 8d depicts the predicted homodimeric C8_DEDs_ complex (red) superposed on the crystallographic structure of the C8_DEDs_ filament (green) (pdb-id: 5L08) with Cα RMSD value of 3.0 Å. The MM-GBSA calculations showed that the free energy of binding of the C8_DEDs_–C8_DEDs_ complex is −71.9 kcal mol^–1^. The key hot spot residues, identified using alanine scanning calculations, are listed in Table 5 and shown in Figure S13. As Table 5 indicates, residues F122 in the FL motif of C8_1,DED2_ and Y8 in the α1/α4 hydrophobic pocket of C8_2,DED1_ (Figure 6a) have the strongest contribution to the C8_1,DEDs_–C8_2,DEDs_ binding affinity.

**Table 5:** Residues with the Highest Contributions to the C8_1,DEDs_–C8_2,DEDs_ Binding Affinity Identified by Alanine Scanning Calculations^a^

mutation in C8_1_	ΔAff kcal mol^–1^	mutation in C8_2_	ΔAff kcal mol^–1^
F122A	20.3	Y8A	14.6
R118A	8.4	F3A	11.8
		R5A	11.8
		R52A	11.0
		M1A	9.4
		Q46A	9.3
		Q49A	8.5
		K39A	5.2

a Only mutated residues with ΔAff > 5 kcal mol^–1^ are listed.

Figure 8e shows the Kelley penalty plot for the C8_DEDs_–cFLIP_DEDs_ docking and clustering calculations, which implies that the optimum number of clusters is 20. The associated distance matrix is presented in Figure 8f. The most populated cluster with 45 members is illustrated in Figure 8g. The standard deviation, population, and average RMSD from the centroid of each cluster along with the relative RMSD values between the centroid of each cluster and the centroid of the most populated one (as reference) are presented in Figure S7f. Figure 8h shows the predicted C8_DEDs_–cFLIP_DEDs_ complex from the protein–protein docking calculations (red) superposed on the crystallographic structure of the C8_DEDs_ filament (green) (pdb-id: 5L08) with Cα RMSD value of 3.4 Å. The MM-GBSA calculations showed that the free energy of binding of the C8_DEDs_–cFLIP_DEDs_ complex is −68.8 kcal mol^–1^, similar to the C8_DEDs_–C8_DEDs_ and FADD_DED_–C8_DEDs_ interaction energies. The key hot spot residues engaging in the complex formation were also identified using alanine scanning calculations and are listed in Table 6 and shown in Figure S14.

**Table 6:** Residues with the Highest Contributions to the C8_DEDs_–cFLIP_DEDs_ Binding Affinity Identified by Alanine Scanning Calculations^a^

mutation in C8	ΔAff kcal mol^–1^	mutation in cFLIP	ΔAff kcal mol^–1^
F122A	12.0	R45A	10.8
R118A	7.9	R47A	9.3
Q125A	5.1	L41A	5.5
		D39A	5.3

a Only mutated residues with ΔAff > 5 kcal mol^–1^ are listed.

The stabilities of the predicted structures of C8_DEDs_ in complex with C8_DEDs_ or cFLIP_DEDs_ were validated using MD simulations. Figures 8i–l shows the Cα RMSD and RMSF of the complexes during the 300 ns MD simulations. As the figure shows, the predicted complexes remain stable during the MD simulations. However, the RFMS plots indicate that the fluctuations of the cFLIP_DEDs_ molecule are larger than those of C8_DEDs_ in the C8_DEDs_–cFLIP_DEDs_ complex. To address this observation, we measured the number of interactions (hydrogen bonds and salt bridges) within each molecule during the MD simulation. As Figure S16 shows, while the average number of salt bridge interactions within C8_DEDs_ and cFLIP_DEDs_ molecules are almost the same (19 vs. 20), the average number of hydrogen bonds are particularly different (184 vs 171) which counted to more than one hydrogen bond per residue for C8_DEDs_ and less than one hydrogen bond per residue for cFLIP_DEDs_. This could be a reason behind the larger fluctuation of the cFLIP_DEDs_ molecule compared with C8_DEDs_.

### Cluster Component Analysis

To assess the docking performance of our new meta-approach, individual clustering component analysis has been performed with each docking engine used in this study (HADDOCK, ClusPro, HDOCK, GRAMM-X, and ZDOCK). As mentioned in the Materials and Methods section, the top five predicted complexes (i.e., *X*_1_–*X*_5_ in Figure 3) from each docking engine were chosen and refined using two different relaxation protocols. In the first protocol only distance restraints were applied, while the second protocol applied both distance and position restraints. Finally, the five lowest energy complexes from each refinement protocol were returned. Therefore, *X_m_n* (*X* = HADDOCK, ClusPro, HDOCK, GRAMM-X, and ZDOCK, *m* = 1–5, *n* = 1–10) represents the model *m*th predicted by docking engine *X* which was refined by the first (*n* = 1–5) and second (*n* = 6–10) relaxation protocols.

In Fas_DD_–Fas_DD_ complex, the most populated cluster consists of 83 members (Figure 4f). The contribution of each docking engines in the main cluster (cluster 7 in Figure S7a) is shown in Figure S17a. ZDOCK, CLusPro, HDOCK, HADDOCK, and GRAMM-X contribute with 31 (∼37%), 15 (∼18%), 15 (∼18%), 13 (∼16%), and 9 (∼11%) members, respectively. The nearest component to centroid of the cluster is ZDOCK_3_6. Table S4 shows the components of each model in the main cluster. In the Fas_DD_–FADD_DD_ complex, the two most populated clusters, with 61 and 50 members, correspond to the Fas_1,DD_–FADD_1,DD_ and Fas_2,DD_–FADD_2,DD_ interactions, respectively (Figure 5c). The contribution of each docking engines in the main clusters (clusters 15 and 9 in Figure S7b) are shown in Figure S17b and c, respectively. ZDOCK, HADDOCK, HDOCK, and GRAMM-X contribute with 20 (∼33%), 20 (∼33%), 10 (∼17%), and 10 (∼17%) members in cluster 15, respectively. The nearest component to the centroid of cluster 15 is GRAMM-X_2_8. On the other hand, ZDOCK, HDOCK, and GRAMM-X contribute with 30 (∼60%), 10 (∼20%), and 10 (∼20%) members in cluster 9, respectively. The nearest component to the centroid of cluster 9 is GRAMM-X_1_1. Tables S5 and S6 show the components of each model in clusters 15 and 9, respectively.

In the FADD_DED_–C8_DEDs_ complex, the most populated cluster consists of 70 members (Figure 7c). The contribution of each docking engine in the main cluster (clusters 3 in Figure S7c) is shown in Figure S17d. ClusPro, GRAMM-X, and ZDOCK contribute with 30 (∼43%), 20 (∼29%), and 20 (∼29%) members, respectively. The nearest component to the centroid of the main cluster is ZDOCK_4_2. In the FADD_DED_–cFLIP_DEDs_ complex, the most populated cluster consists of 30 members (Figure 7g). The contribution of each docking engine in the main cluster (clusters 3 in Figure S7d) is shown in Figure S17e. ClusPro, GRAMM-X, and HDOCK contribute equally with 10 (∼33%) members each. The nearest component to the centroid of the cluster is HDOCK_4_10. Tables S7 and S8 show the components of each model in the main clusters of FADD_DED_–C8_DED_ and FADD_DED_–cFLIP_DED_ complexes, respectively.

In the C8_DEDs_–C8_DEDs_ complex, the most populated cluster consists of 57 members (Figure 8c). The contribution of each docking engine in the main cluster (clusters 24 in Figure S7e) is shown in Figure S17f. ClusPro, GRAMM-X, HDOCK, and HADDOCK contribute with 20 (∼35%), 18 (∼32%), 10 (∼18%), and 9 (∼16%) members, respectively. The nearest component to the centroid of the main cluster is HADDOCK_2_1. In the C8_DEDs_–cFLIP_DEDs_ complex, the most populated cluster consists of 45 members (Figure 8g). The contribution of each docking engines in the main cluster (clusters 6 in Figure S7f) is shown in Figure S17g. HADDOCK, GRAMM-X, and HDOCK contribute with 20 (∼44%), 20 (∼44%), and 5 (∼11%) members, respectively. The nearest component to the centroid of the cluster is HDOCK_3_5. Tables S9 and S10 show the components of each model in the main clusters of C8_DEDs_–C8_DEDs_ and C8_DEDs_–cFLIP_DEDs_ complexes, respectively.

The clustering component analysis clearly shows that it is difficult to identify the best docking pose in PP docking using just one docking engine. For example, ZDOCK shows big contributions in the main cluster of Fas_DD_–Fas_DD_ and Fas_DD_–FADD_DD_ complexes, less contribution in the FADD_DED_–C8_DEDs_ complex, and no contribution in other complexes. Similarly, ClusPro shows no contribution in Fas_DD_–FADD_DD_ and C8_DEDs_–cFLIP_DEDs_ complexes while it has a prominent contribution in FADD_DED_–C8_DEDs_, FADD_DED_–cFLIP_DEDs_, and C8_DEDs_–C8_DEDs_ complexes. GRAMM-X is the only docking engine which has some members in every clusters. Moreover, it is not easy to determine which model generated by individual docking engine represents the best binding mode of any specific protein complex. The meta-approach introduced in this study could be even more effective if larger numbers of models, generated by each docking engines, are considered for further refinement and clustering.

## Conclusions

Using a meta-approach for protein–protein docking in which we combined the data obtained from the protein–protein docking engines HADDOCK, ClusPro, HDOCK, GRAMM-X, and ZDOCK, the structures of the different dimeric components of the DISC complex were predicted to high accuracy. The computed MM-GBSA interaction energies of each of the most stable complexes are summarized in Table 7. The Fas–Fas and Fas–FADD_DD_ interactions are very strong, which promotes the formation of the DISC core (Figure 1b). Binding to the FADD_DED_ is significantly stronger for C8_DEDs_ than for cFLIP_DEDs_, a fact that was also manifested in the MD simulations (Figure 7). However, the binding energies of C8_DEDs_–C8_DEDs_ and C8_DEDs_–cFLIP_DEDs_ are of similar magnitude and may thus compete in subsequent buildup of the DISC filament. The equal interaction between C8_DEDs_–C8_DEDs_ and C8_DEDs_–cFLIP_DEDs_ was validated in a series of PCA analyses.

**Table 7:** Interaction Energies (MM-GBSA; kcal mol^–1^) of the Identified Most Stable Protein–Protein Complexes

protein–protein complex	interaction energy
Fas_DD_–Fas_DD_	–116.7
Fas_DD_–FADD_DD_	–155.1
FADD_DED_–C8_DEDs_	–60.9
FADD_DED_–cFLIP_DEDs_	–37.0
C8_DEDs_–C8_DEDs_	–71.9
C8_DEDs_–cFLIP_DEDs_	–68.8

**Figure 9:**
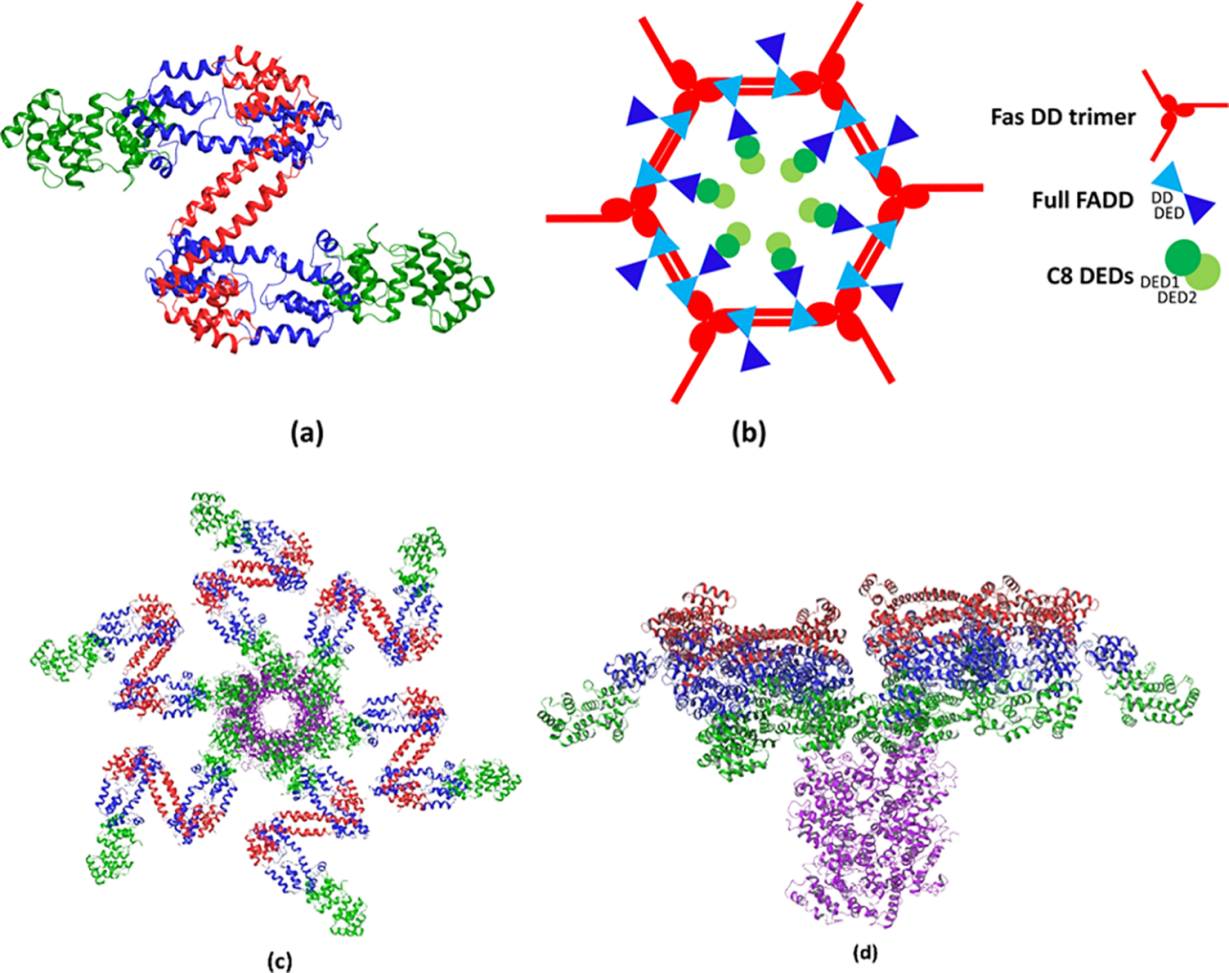
(a) Smallest unit of the DISC which contains 2 Fas_DD_, 2 FADD_full_, and 2 C8_DEDs_ molecules. Fas_DD_s, FADD_full_s, and C8_DEDs_ are presents in red, blue, and green, respectively. (b) FADD_full_ and C8_DEDs_ proteins potentially bind to the hexagonal structure formed by dimerization of the opened Fas_DD_ trimers. (c) Top and (d) side view of the possible architecture of the DISC network in which the C8_DEDs_ DED filament (purple) starts to form through the recruitment of free C8_DEDs_ molecules with those bound to the FADD_DED_ (green).

Based on the *in silico* results of protein–protein docking and MD simulations, we then reconstructed the smallest unit of the DISC which contains 2 Fas_DD_, 2 FADD_full_, and 2 C8_DEDs_ molecules (Figure 9a). To generate this model, the DD of FADD_full_ was first superposed on the DD of FADD in the Fas_DD_–FADD_DD_ complex after which the DED of FADD in the FADD_DED_–C8_DEDs_ complex was superposed on the DED of FADD in the Fas_DD_–FADD_full_ complex. The result illustrated in Figures 9a and b shows how the FADD_full_ and C8_DEDs_ proteins are able to bind in a hexagonal structure formed by dimerization of the opened Fas_DD_ trimers. Based on this model, six C8_DEDs_ bound to the six FADD_DED_ molecules align at the center of the hexagonal ring (*cf*. Figure 1b). Since there is no information or crystallographic data on how Fas trimerizes through the globular part of their DDs, it is difficult to exactly construct the DISC network. However, Figures 9c (top view) and d (side view) show a possible architecture of the DISC network in which a C8_DEDs_ filament (purple) starts to form through the interaction of free C8_DEDs_ molecules with those (in green) bound to the FADD_DED_.

The filament can keep expanding by recruitment of more C8 molecules. Since the structure of the C8_DEDs_ and cFLIP_DEDs_ are highly similar, Fu et al. suggested that cFLIP may comingle with C8.^52^ The incorporation of cFLIP_S,R_ (without the caspase-like domain) into the filament reduces the local concentration of the C8 caspase domain and thus inhibits its dimerization and autoactivation process. On the other hand, the role of cFLIP_L_ in the filament is more complicated. It has been demonstrated that, based on the concentration of cFLIP_L_, it can act either as an antiapoptotic agent, *i*.*e*., reducing the C8 autoactivation at the filament, or as a proapoptotic molecule, *i*.*e*., enhancing the C8 activation.^11^ It has been demonstrated that overexpression of cFLIP in different cancerous cells prohibits the DL-induced apoptosis and makes them resistant against chemotherapy.^53−55^ Therefore, finding and designing small molecules that are capable of selectively targeting c-FLIP and prohibiting its recruitment to the DISC without blocking the formation of the growing C8 filament could be a promising cancer therapy strategy.^56^ The current study provides unique structural information and provides a setting for screening molecular databases in order to find selective bioactive molecules capable of modulating the undesired interactions involving cFLIP within the DISC.

## Supporting Information Available

Table S1 shows a summary of the searching algorithms and scoring functions implemented in the PP docking engines employed in this study. Figures S1–S6 show the quality assessment of the homology models. Table S2 indicates the specified residues in the PP docking procedure. Figure S7 shows statistical information regarding the clusters. Figures S8–S11 and S13–S14 show a close-up view of interacting regions in the protein–protein interaction complexes and placement of the α1/α4 and α2/α5 helices of FADD_DED_ in the Fas_DD_– FADD_full_ complex. Figure S12 shows the native residue contact profiles during 300 ns MD simulation for FADD_DED_–C8_DEDs_ and FADD_DED_–cFLIP_DEDs_ complexes. Table S3 and Figure S15 present information regarding the plasmids used in the PCA analysis. Figure S16 shows the number of hydrogen bonds and salt bridge interactions within C8_DEDs_ and cFLIP_DEDs_ molecules during 300 ns MD simulation. Figure S17 shows the contribution of each docking engines in the main clusters of each PP complex. Tables S4–S10 show the cluster component analysis for the main cluster of each PP complex (PDF)
