# Supplementary material for: Reconstruction of the Fas-Based Death-Inducing Signaling Complex (DISC) Using a Protein–Protein Docking Meta-Approach
Source: J Chem Inf Model. 2021 Jul 1;61(7):3543–58. doi: 10.1021/acs.jcim.1c00301 (PMC8389534; doi:10.1021/acs.jcim.1c00301)
Supplement: Supplementary file 1 — ci1c00301_si_001.pdf [file ci1c00301_si_001.pdf]

Supporting Information

Reconstruction of the Fas-Based Death Inducing Signaling Complex (DISC) Using a Protein-Protein Docking Meta-Approach

Sayyed Jalil Mahdizadeh[1], Melissa Thomas[1] and Leif A. Eriksson[1,*]

[1]Department of Chemistry and Molecular Biology, University of Gothenburg, 405 30 Göteborg, Sweden

Correspondence:

*Leif A. Eriksson, Department of Chemistry and Molecular Biology,

University of Gothenburg, 405 30 Göteborg, Sweden.

Email: leif.eriksson@chem.gu.se

Phone: +46 317869117

Table S1	P2	Figure S13	P13
Figure S1	P2	Figure S14	P13
Figure S2	P3	Table S3	P14
Figure S3	P4	Figure S15	P14
Figure S4	P5	Figure S16	P15
Figure S5	P6	Figure S17	P16
Figure S6	P7	Table S4	P16
Table S2	P7	Table S5	P17
Figure S7	P9	Table S6	P17
Figure S8	P10	Table S7	P17
Figure S8	P10	Table S8	P17
Figure S10	P11	Table S9	P17
Figure S11	P11	Table S10	P17
Figure S12	P12	References	P18

Table S1. Summary of the searching algorithms and scoring functions implemented in the PP docking engines employed in this study.

Docking engine	Searching algorithm	Scoring function	URL	Reference
**HADDOCK**	Flexible Monte Carlo search	Van der Waals, electrostatics, binding site restriction, and buried surface area	https://wenmr.science.uu.nl/	1, 2
**ClusPro**	Rigid body Fast Fourier transform (FFT)	Shape complementarity, electrostatics and desolvation energy	https://cluspro.bu.edu/	3, 4
**HDOCK**	Rigid body FFT. template-based and ab initio template-free docking	Long range shape complementarity and statistical mechanics-based	http://hdock.phys.hust.edu.cn/	5, 6
**GRAMM-X**	Rigid body FFT	shape complementarity and hydrophobicity	http://vakser.compbio.ku.edu/resources/gramm/grammx/	7
**ZDOCK**	Rigid body FFT	shape complementarity, Van der Waals, electrostatics, desolvation energy	https://zdock.umassmed.edu/	8, 9

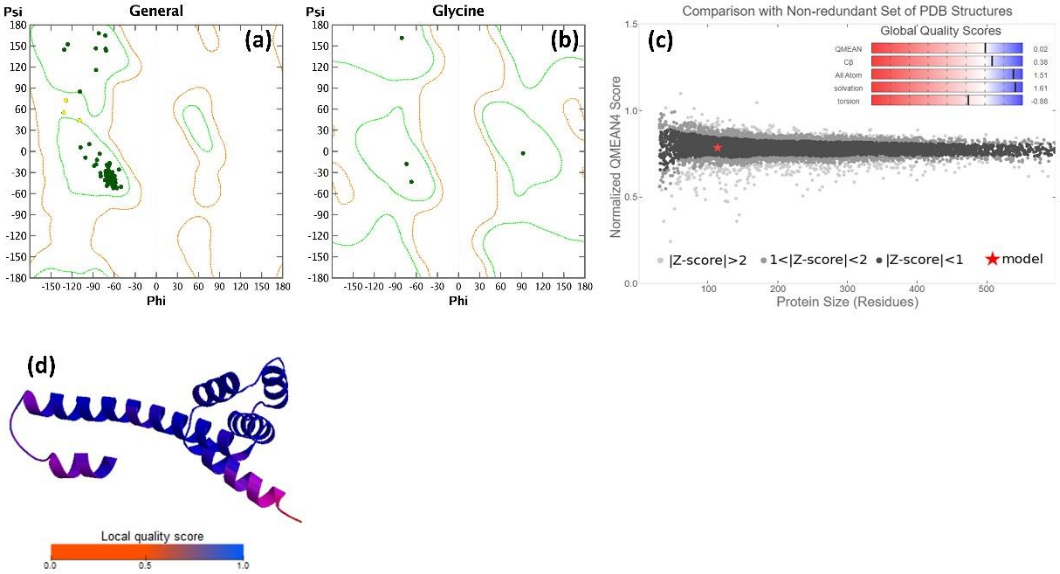

Figure S1. Structural quality assessment for the homology model of DR Fas$_{DD}$. Ramachandran plots of (a) General and (b) Glycine residues. The green and yellow circles reperesent the residues located in core and allowed regions, respectively. (c) Global quality estimation of the model through comparison of the normalized QMEAN4 scores (marked by red asterisk) with a non-redundant set of pdb structures of similar size. The QMEAN score of the model and its components (Cβ, all-atom, solvation, and torsion) are shown as inset. (d) Local quality estimation based on the residue QMEAN score values.

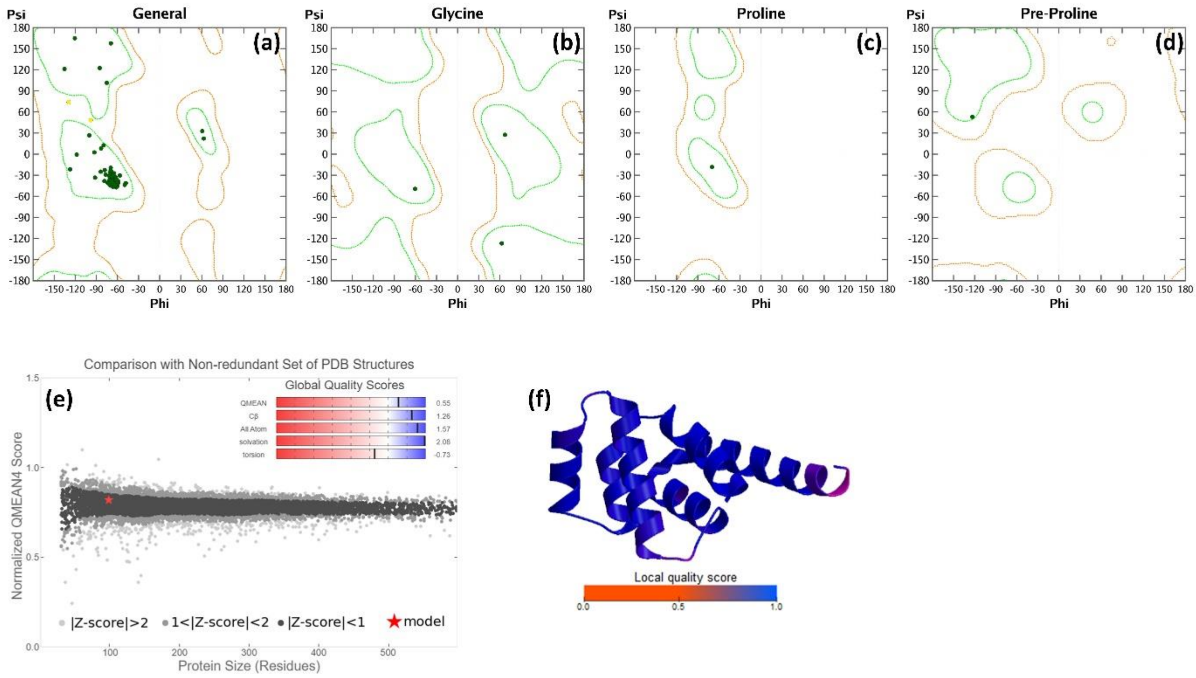

Figure S2. Structural quality assessment for the homology model of $FADD_{DD}$. Ramachandran plots of (a) General, (b) Glycine, (c) Proline, and (d) Pre-Proline residues. The green and yellow circles reperesent the residues located in core and allowed regions, respectively. (e) Global quality estimation of the model through comparison of the normalized QMEAN4 scores (marked by red asterisk) with a non-redundant set of pdb structures of similar size. The QMEAN score of the model and its components (Cβ, all-atom, solvation, and torsion) are shown as inset. (f) Local quality estimation based on the residue QMEAN score values.

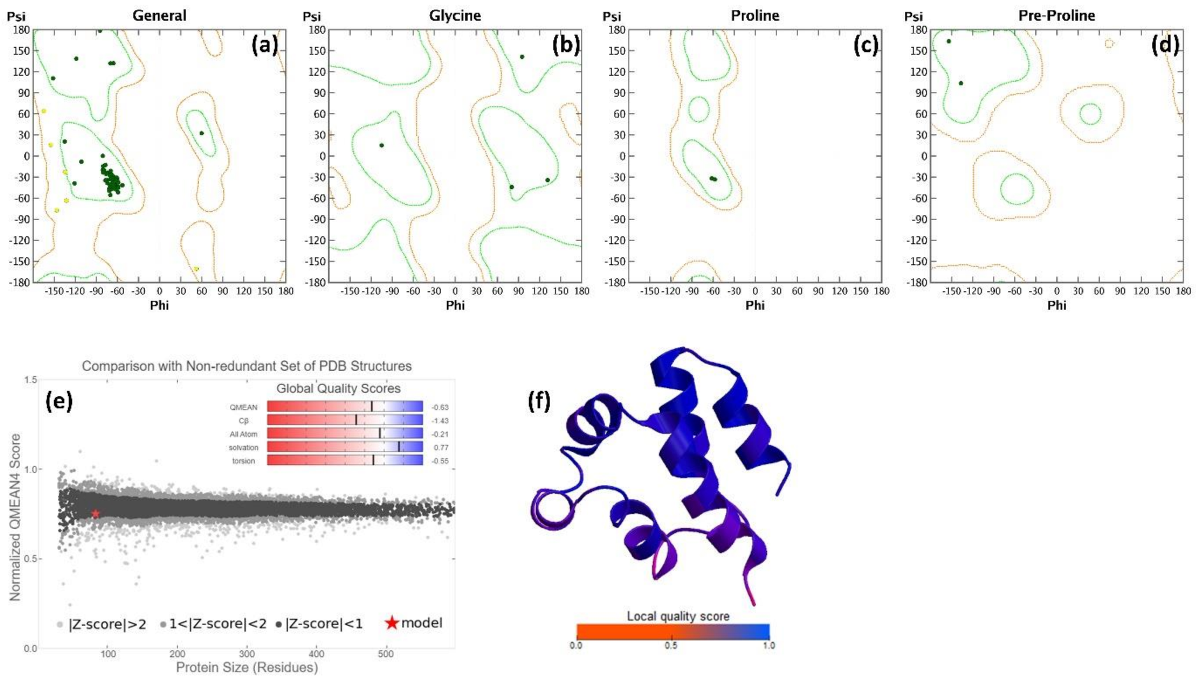

Figure S3. Structural quality assessment for the homology model of $FADD_{DED}$. Ramachandran plots of (a) General, (b) Glycine, (c) Proline, and (d) Pre-Proline residues. The green and yellow circles reperesent the residues located in core and allowed regions, respectively. (e) Global quality estimation of the model through comparison of the normalized QMEAN4 scores (marked by red asterisk) a non-redundant set of pdb structures of similar size. The QMEAN score of the model and its components (Cβ, all-atom, solvation, and torsion) are shown as inset. (f) Local quality estimation based on the residue QMEAN score values.

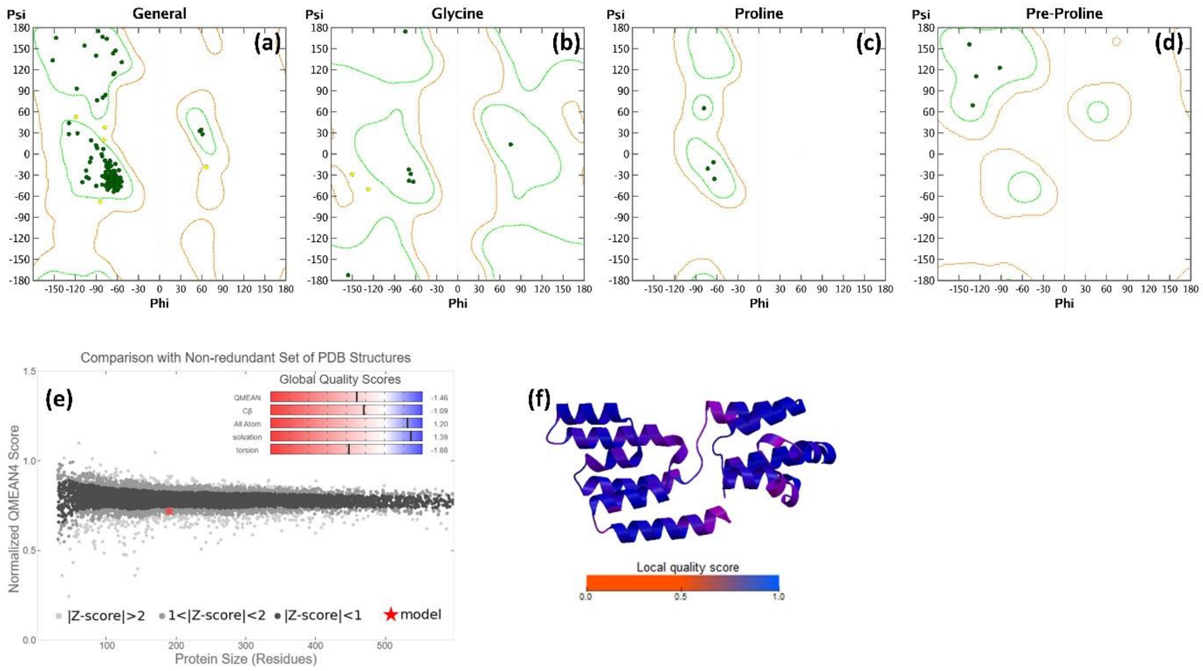

Figure S4. Structural quality assessment for the homology model of $FADD_{full}$. Ramachandran plots of (a) General, (b) Glycine, (c) Proline, and (d) Pre-Proline residues. The green and yellow circles reperesent the residues located in core and allowed regions, respectively. (e) Global quality estimation of the model through comparison of normalized QMEAN4 scores (marked by red asterisk) with a non-redundant set of pdb structures of similar size. The QMEAN score of the model and its components (Cβ, all-atom, solvation, and torsion) are shown as inset. (f) Local quality estimation based on the residue QMEAN score values.

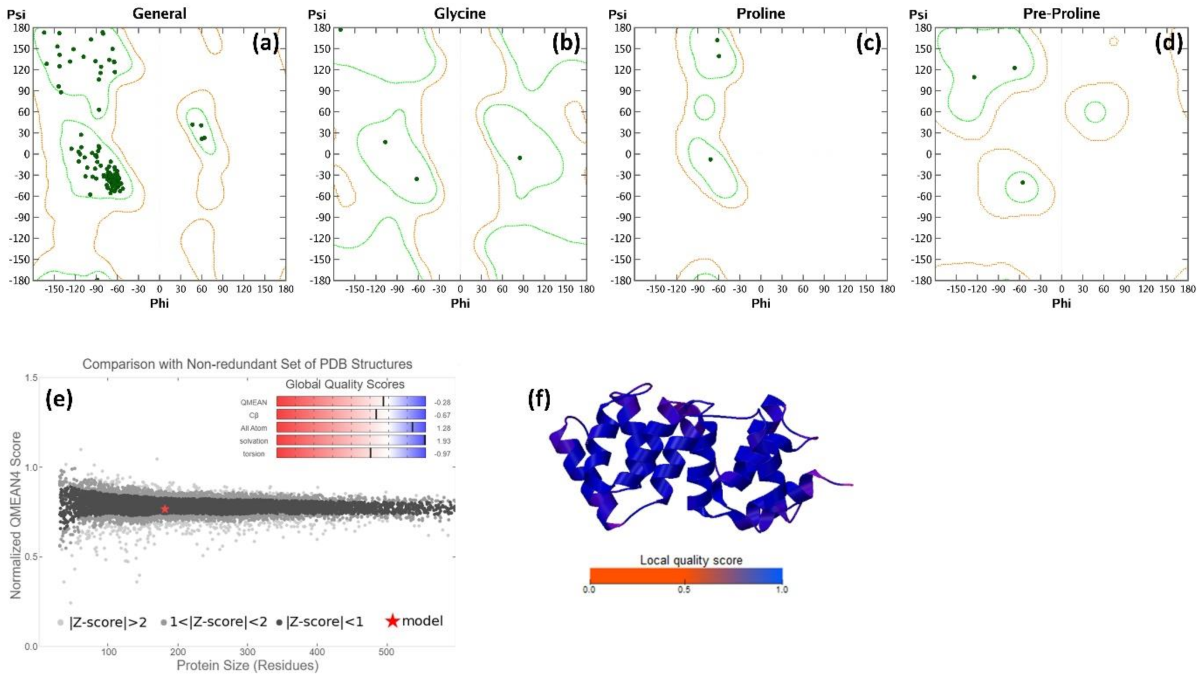

Figure S5. Structural quality assessment for the homology model of $C8_{DEDs}$. Ramachandran plots of (a) General, (b) Glycine, (c) Proline, and (d) Pre-Proline residues. The green and yellow circles reperesent the residues located in core and allowed regions, respectively. (e) Global quality estimation of the model through comparison of the normalized QMEAN4 scores (marked by red asterisk) with a non-redundant set of pdb structures of similar size. The QMEAN score of the model and its components (Cβ, all-atom, solvation, and torsion) are shown as inset. (f) Local quality estimation based on the residue QMEAN score values.

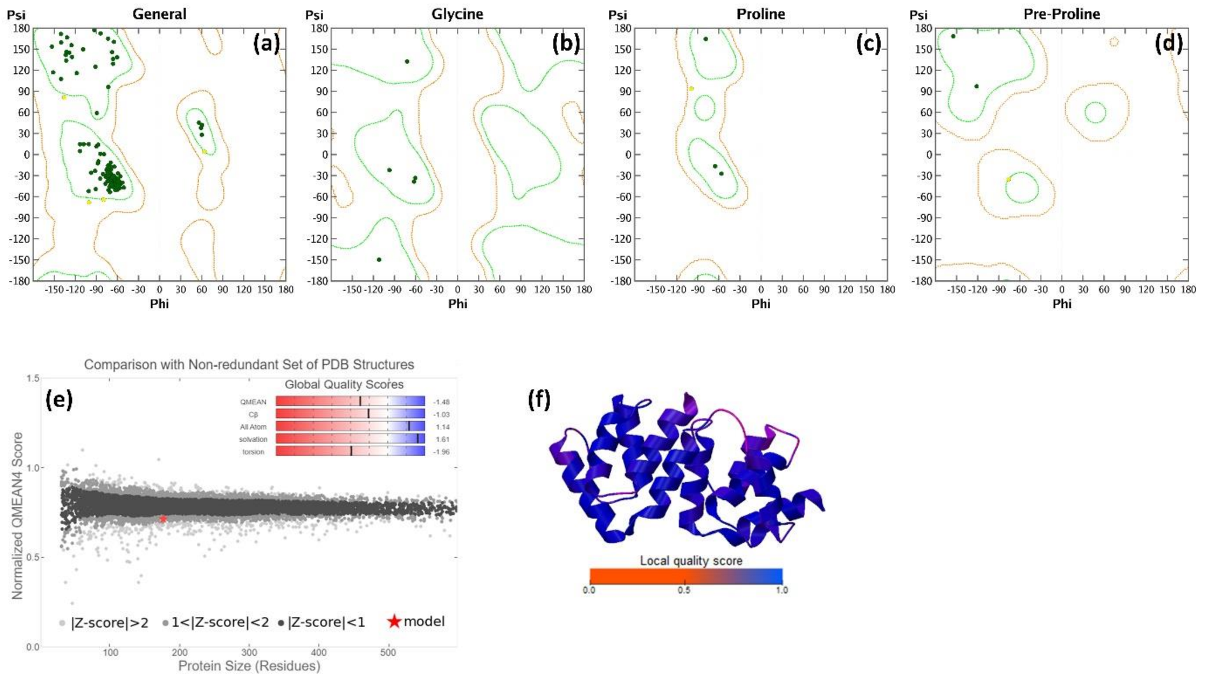

Figure S6. Structural quality assessment for the homology model of cFLIP$_{DEDs}$. Ramachandran plots of (a) General, (b) Glycine, (c) Proline, and (d) Pre-Proline residues. The green and yellow circles reperesent the residues located in core and allowed regions, respectively. (e) Global quality estimation of the model through comparison of the normalized QMEAN4 scores (marked by red asterisk) with a non-redundant set of pdb structures of similar size. The QMEAN score of the model and its components (Cβ, all-atom, solvation, and torsion) are shown as inset. (f) Local quality estimation based on the residue QMEAN score values.

Table S2. The specified residues in PP docking procedure.

PP complex	Components	Residues
**Fas$_{DD}$-Fas$_{DD}$**	Fas$_{DD}$	K299, I310
	Fas$_{DD}$	K299, I310
**Fas$_{DD}$-FADD$_{DD}$**	Fas$_{DD}$	Y232, T235, I295, L298
	FADD$_{DD}$	L172, L176, L186
**FADD$_{DED}$-C8$_{DEDs}$**	FADD$_{DED}$	F25, L26
	C8$_{DEDs}$	Y8
**FADD$_{DED}$-cFLIP$_{DEDs}$**	FADD$_{DED}$	F25, L26
	cFLIP$_{DEDs}$	H7
**C8$_{DEDs}$-C8$_{DEDs}$**	C8$_{DEDs}$	F122, L123
	C8$_{DEDs}$	Y8
**C8$_{DEDs}$-cFLIP$_{DEDs}$**	C8$_{DEDs}$	F122, L123
	cFLIP$_{DEDs}$	H7

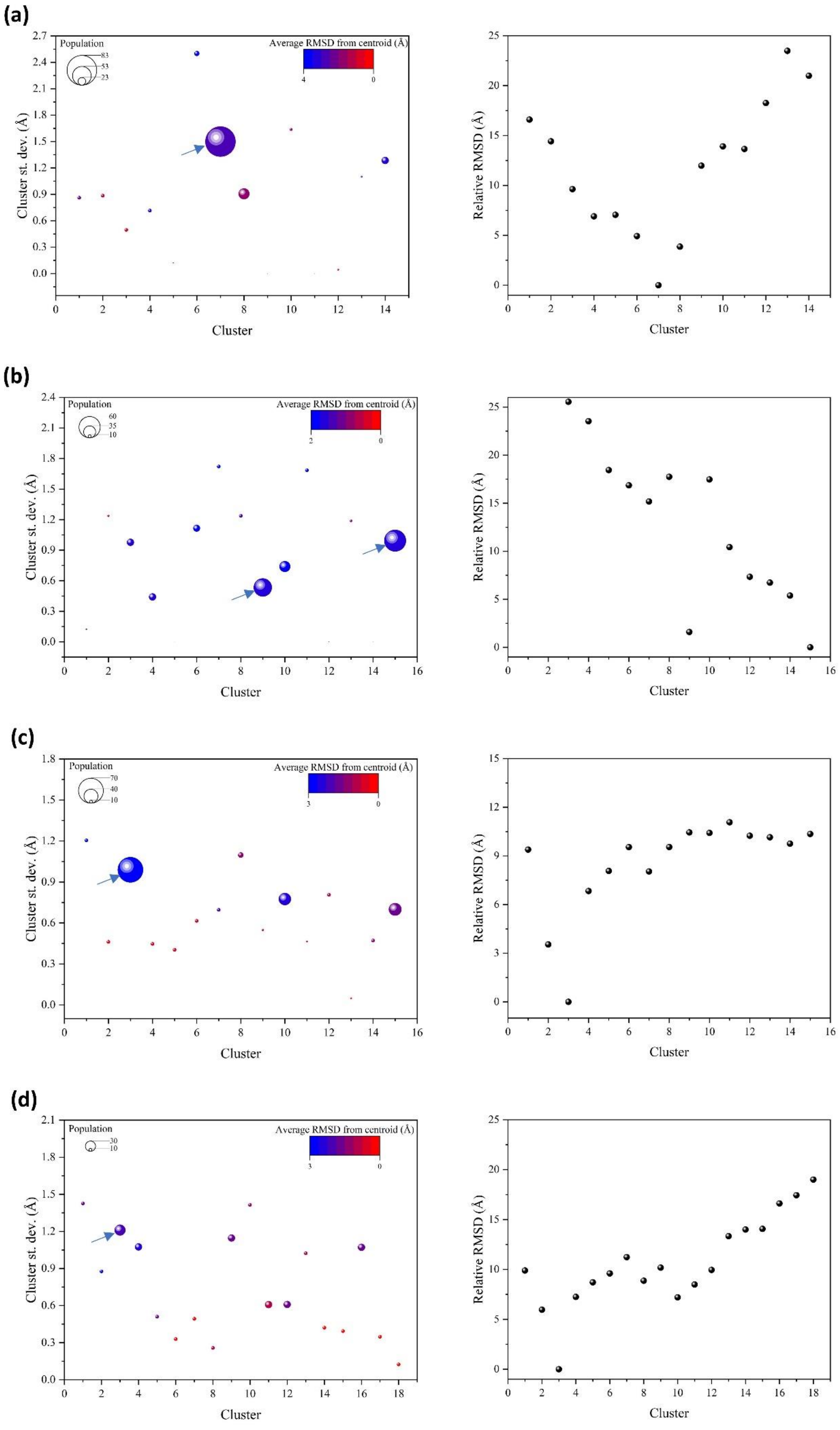

(a)
(b)
(c)
(d)
Population
Average RMSD from centroid (Å)
Cluster st. dev. (Å)
Relative RMSD (Å)
Cluster

(e)

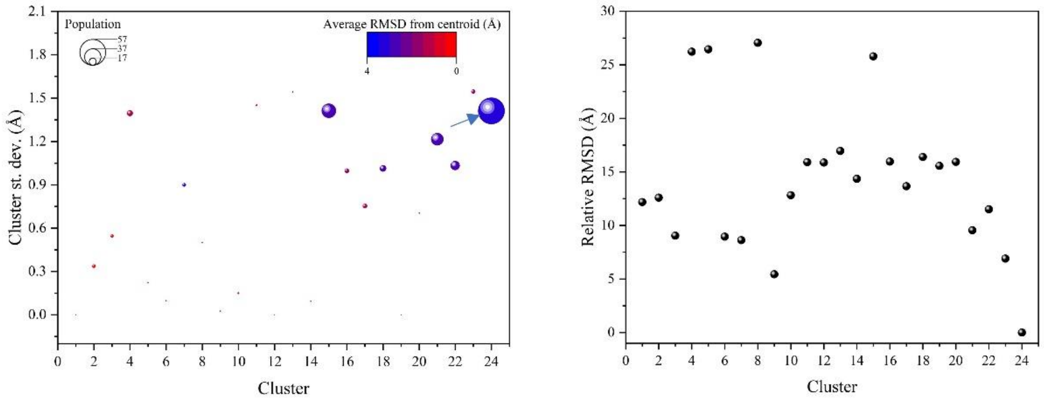

(f)

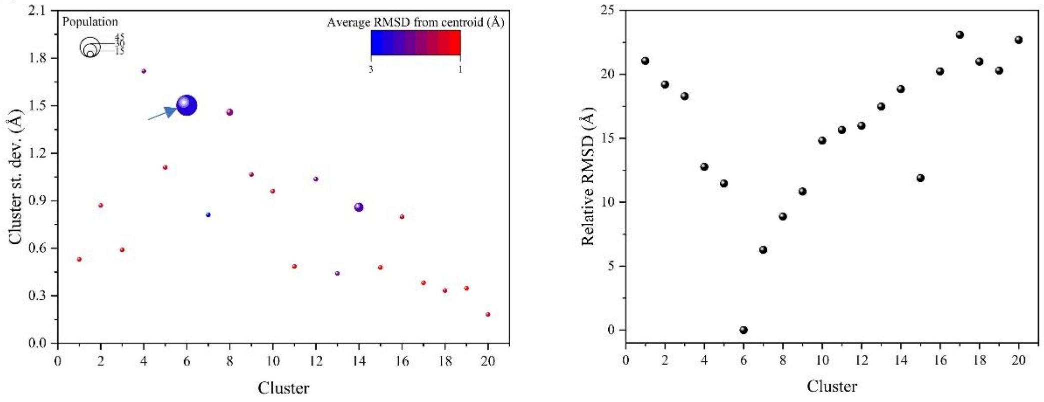

Figure S7. Left hand panels: The standard deviation (y-axis), population (sphere size), and average RMSD (coloration) from the centroid within each cluster. Right hand panels: Relative RMSD values between the centroid of each cluster and the centroid of the most populated one. (a) Fas$_{DD}$–Fas$_{DD}$, (b) Fas$_{DD}$–FADD$_{DD}$, (c) FADD$_{DED}$–C8$_{DEDs}$, (d) FADD$_{DED}$–cFLIP$_{DEDs}$, (e) C8$_{DEDs}$–C8$_{DEDs}$, and (f) C8$_{DEDs}$–cFLIP$_{DEDs}$. The most populated clusters are indicated by small arrows.

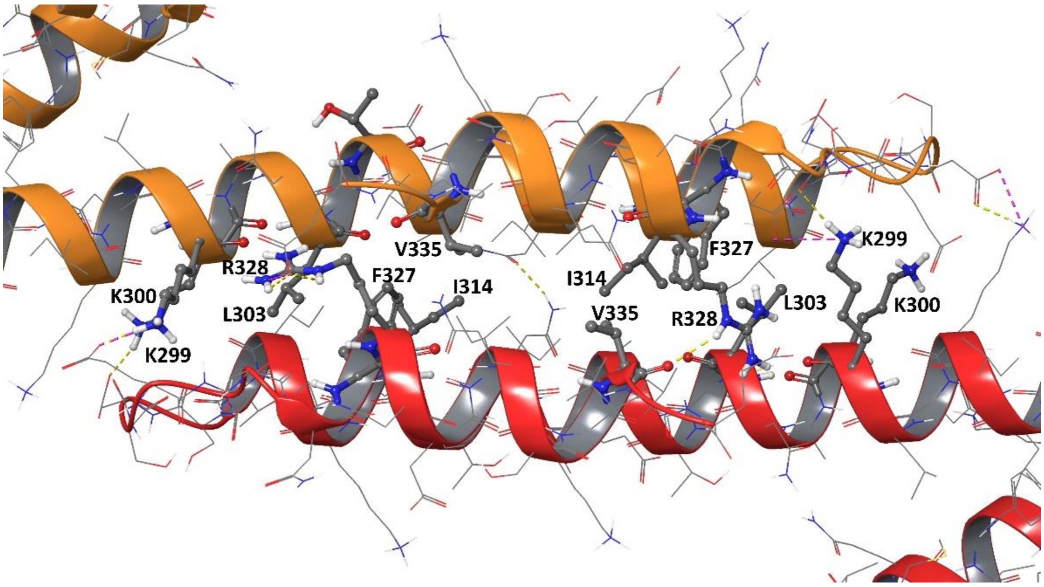

Figure S8. Close-up view of atomic contacts in the Fas_{DD}–Fas_{DD} complex. The $Fas_{1,DD}$ and $Fas_{2,DD}$ chains are presented in red and orange colors, respectively. Key interacting residues (Table1) are presented in ball-and-stick representation. Hydrogen atoms bound to carbon atoms are omitted for clarity.

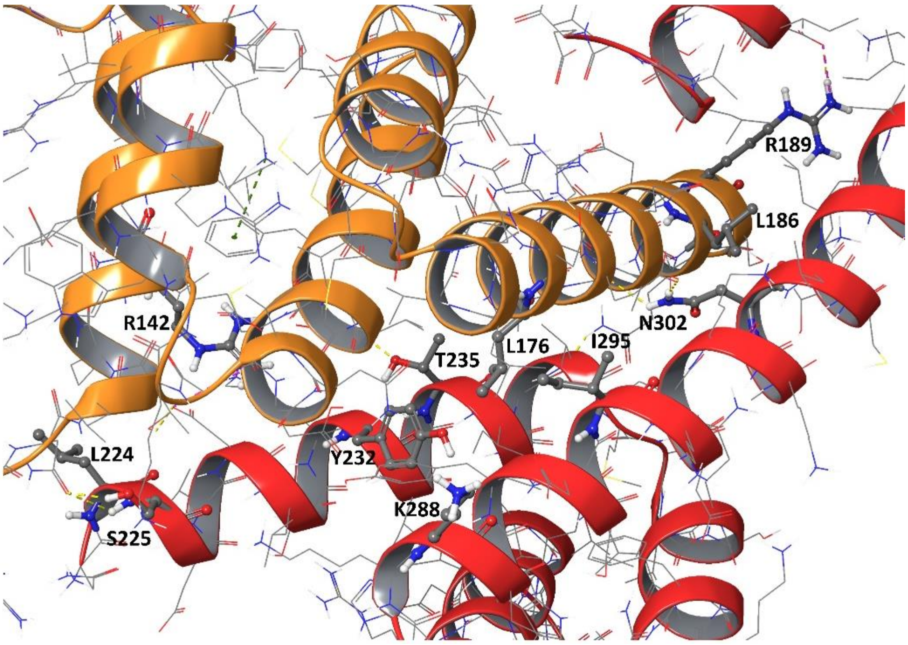

Figure S9. Close-up view of atomic contacts in the Fas_{DD}–$FADD_{DD}$ complex. The Fas_{DD} and $FADD_{DD}$ chains are presented in red and orange colors, respectively. Key interacting residues (Table2) are presented in ball-and-stick representation. Hydrogen atoms bound to carbon atoms are omitted for clarity.

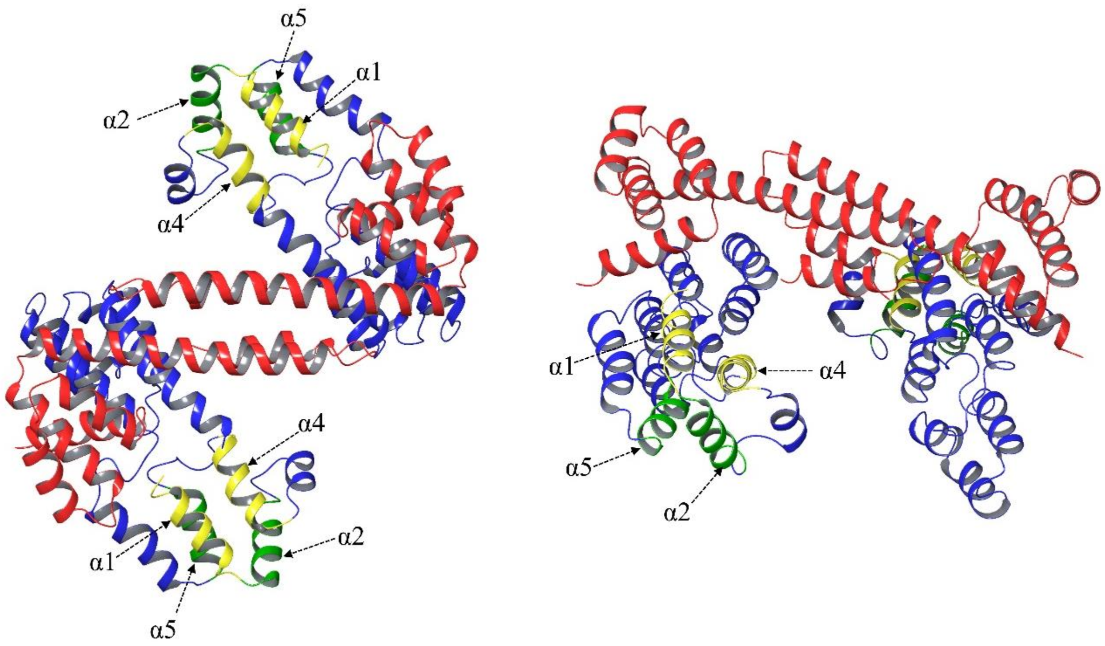

Figure S10. Top (left) and side (right) views of α1/α4 (in yellow) and α2/α5 (in green) helices of $FADD_{DED}$ in the Fas_{DD}–$FADD_{full}$ complex. The Fas_{DD} and $FADD_{full}$ are represented in red and blue colors, respectively.

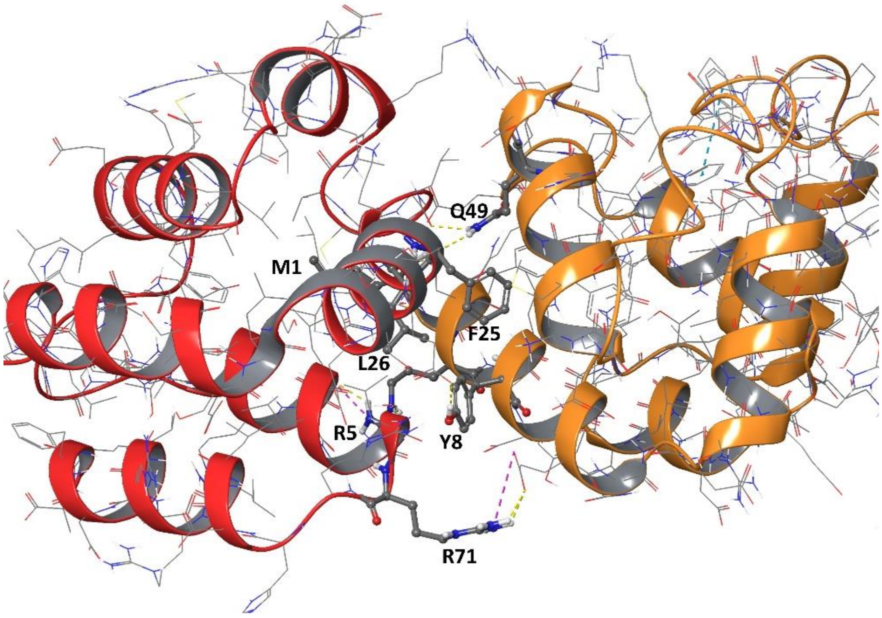

Figure S11. Close-up view of atomic contacts in the $FADD_{DED}$–$C8_{DED}$ complex. The $FADD_{DED}$ and $C8_{DED}$ chains are presented in red and orange colors, respectively. Key interacting residues (Table3) are presented in ball-and-stick representation. Hydrogen atoms bound to carbon atoms are omitted for clarity.

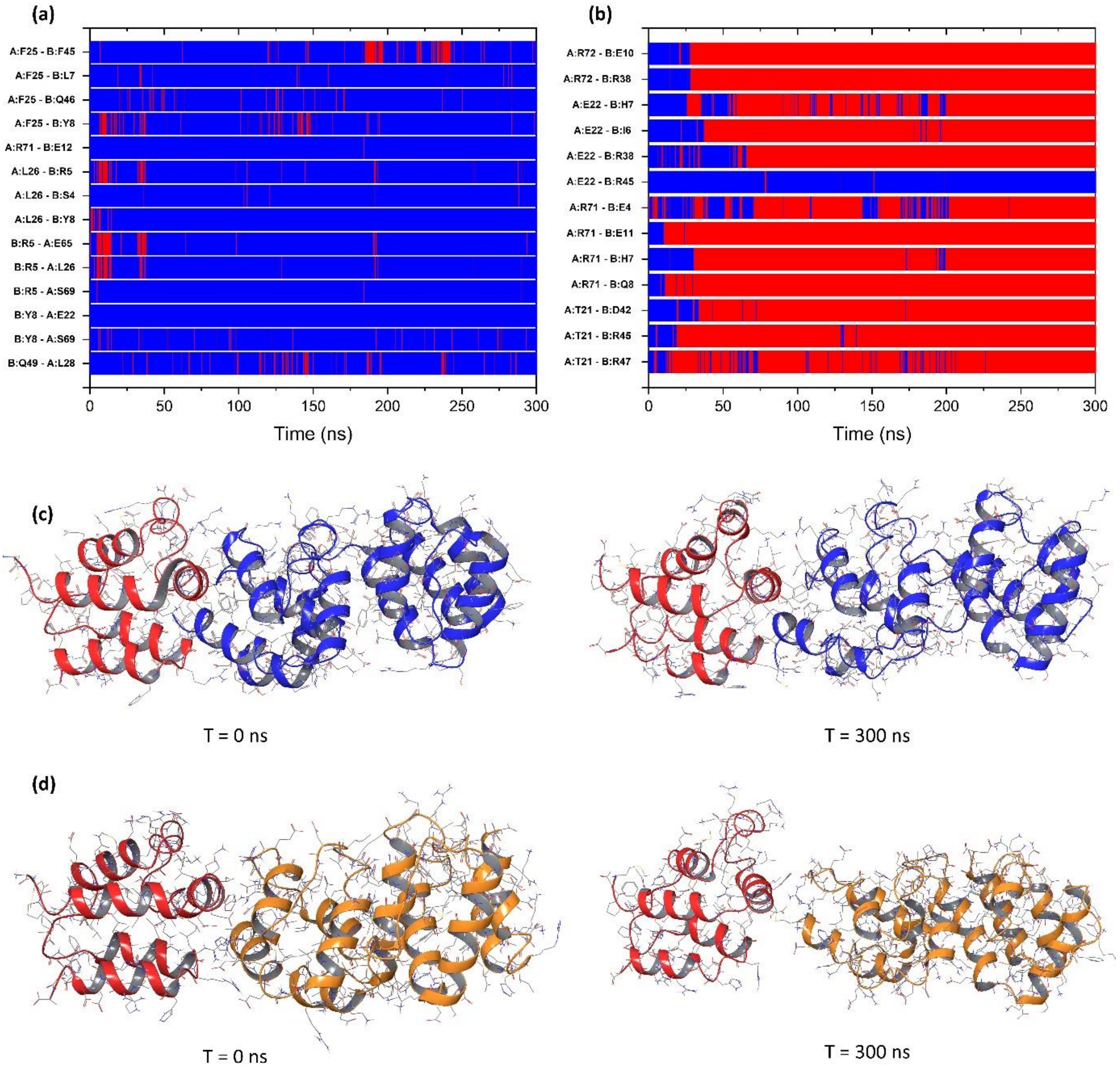

Figure S12. The native residue contacts profiles during 300 ns MD simulation for (a) FADD$_{DED}$–C8$_{DEDs}$ and (b) FADD$_{DED}$–cFLIP$_{DEDs}$ complexes. The blue and red colors indicate that the specific native contact was maintained or diminished, respectively. Chain A and B represent FADD$_{DED}$ and C8$_{DEDs}$/cFLIP$_{DEDs}$ molecules, respectively. The first (t= 0 ns) and last (t = 300 ns) snapshots of MD trajectory for (a) FADD$_{DED}$–C8$_{DEDs}$ and (b) FADD$_{DED}$–cFLIP$_{DEDs}$ complexes. FADD$_{DED}$ is shown by red ribbon while C8$_{DEDs}$ and cFLIP$_{DEDs}$ are presented by blue and orange ribbons, respectively.

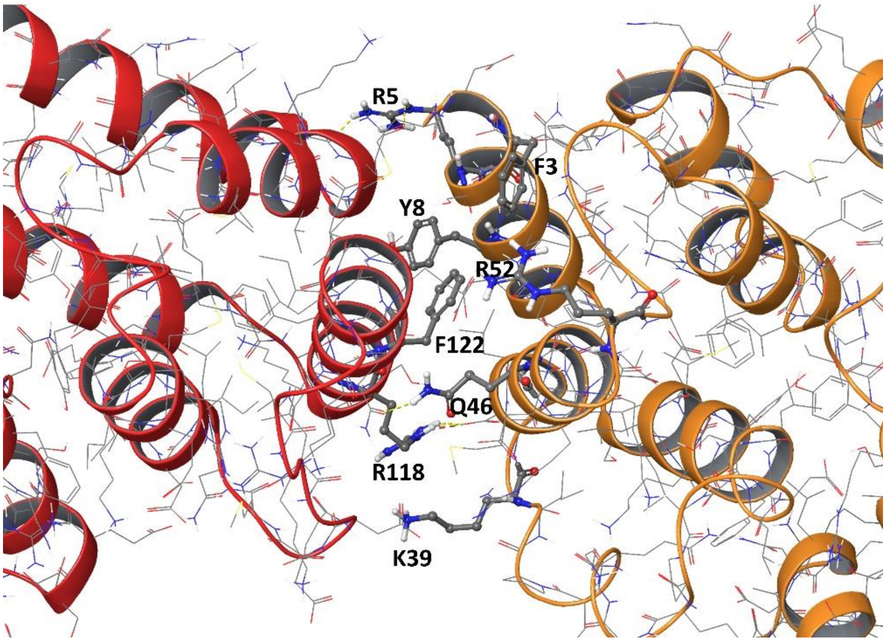

Figure S13. Close-up view of atomic contacts in the C8$_{DEDs}$–C8$_{DEDs}$ complex. The C8$_{1,DEDs}$ and C8$_{2,DEDs}$ chains are presented in red and orange colors, respectively. Key interacting residues (Table 5) are presented in ball-and-stick representation. Hydrogen atoms bound to carbon atoms are omitted for clarity.

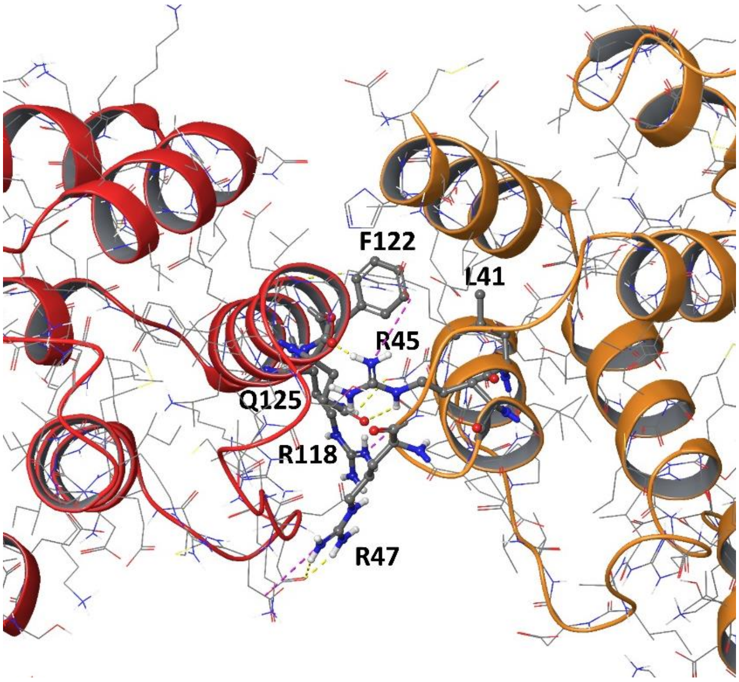

Figure S14. Close-up view of atomic contacts in the C8$_{DEDs}$–cFLIP$_{DEDs}$ complex. The C8$_{DEDs}$ and cFLIP$_{DEDs}$ chains are presented in red and orange colors, respectively. Key interacting residues (Table 6) are presented in ball-and-stick representation. Hydrogen atoms bound to carbon atoms are omitted for clarity.

Table S3: Table of the primers used to clone the different inserts in the Linker-hRluc-F(1)_pcDNA3.1(+) and Linker-hRluc-F(2)_pcDNA3.1(+) and the name of the resulting fusion proteins.[a]

Plasmid name	Primers (5' – 3')		Coding protein	Renilla Luc domain
	Forward	Reverse		
Caspase8-DED1_linker_hRLuc-F(1)_pcDNA3.1(+)	TAGTCCAGTGTGGTGGAATTCGCCACCATGGA CTTCAGCAGAAATCTTTATG	TCCAGAGCCACCGCCACCATCGATGTTTAGGTAGG TAATCAGCAAATC	Caspase 8 death effector domain 1	Nterm-RLUC
Caspase8-DED2_linker_hRLuc-F(1)_pcDNA3.1(+)	TAGTCCAGTGTGGTGGAATTCGCCACCATG GCCTACAGGGTCATGCTCTA	TCCAGAGCCACCGCCACCATCGATGTCGTTGATTAT CTTCAGCAG	Caspase 8 death effector domain 2	Nterm-RLUC
FLIP_linker_hRLuc_F(1)_pcDNA3.1(+)	TAGTCCAGTGTGGTGGAATTCGCCACCATGTCT GCTGAAGTCATCCATC	TCCAGAGCCACCGCCACCATCGATTGTGTAGGAGA GGATAAGTTTC	cFLIP whole protein	Nterm-RLUC
FLIP_linker_hRLuc_F(1)_pcDNA3.1(+)	TAGTCCAGTGTGGTGGAATTCGCCACCATGTCT GCTGAAGTCATCCATC	TCCAGAGCCACCGCCACCATCGATTGTGTAGGAGA GGATAAGTTTC	cFLIP whole protein	Cterm-RLUC
FLIP-DED1_linker_hRLuc-F(2)_pcDNA3.1(+)	TAGTCCAGTGTGGTGGAATTCGCCACCATGTCT GCTGAAGTCATCCATC	TCCAGAGCCACCGCCACCATCGATCTTCAAGATAC GTTTGAGCAGG	cFLIP death effector domain 1	Cterm-RLUC
FLIP-DED2_linker_hRLuc-F(2)_pcDNA3.1(+)	TAGTCCAGTGTGGTGGAATTCGCCACCATG GACTATAGAGTGCTGATGGC	TCCAGAGCCACCGCCACCATCGATCTTCTGGATTTT TGTCTTCAGG	cFLIP death effector domain 2	Cterm-RLUC

[a]*The underlined parts of the primer sequence indicate the complementary area that hybridize to the gene of interest.*

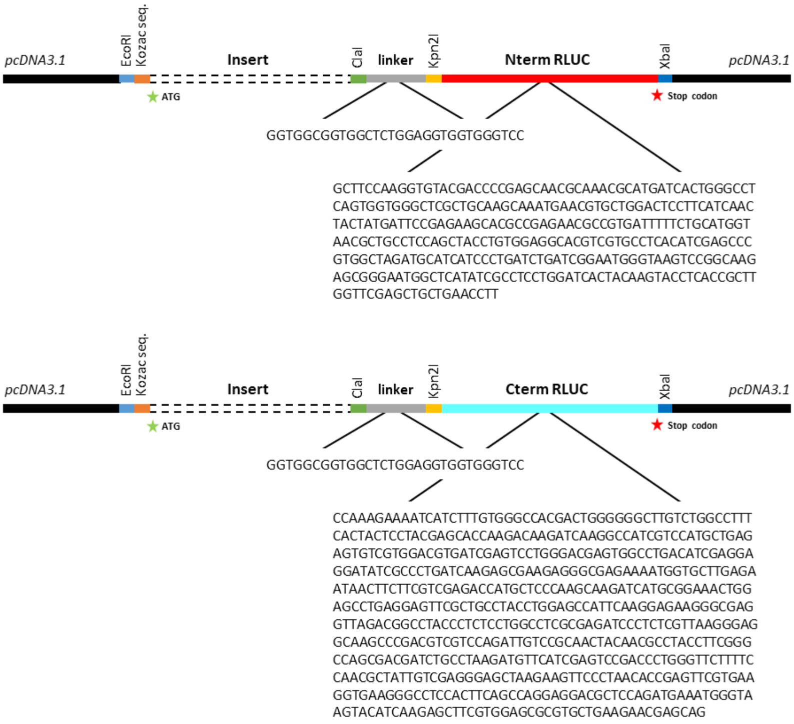

Figure S15. Schematic representation of the Linker-hRluc-F(1)_pcDNA3.1(+) and Linker-hRluc-F(2)_pcDNA3.1(+) plasmid coding parts. In both cases, the shortest sequence represents the linker, and the longer one represents the Nterm and Cterm Renilla Luciferase sequence fragments, respectively.

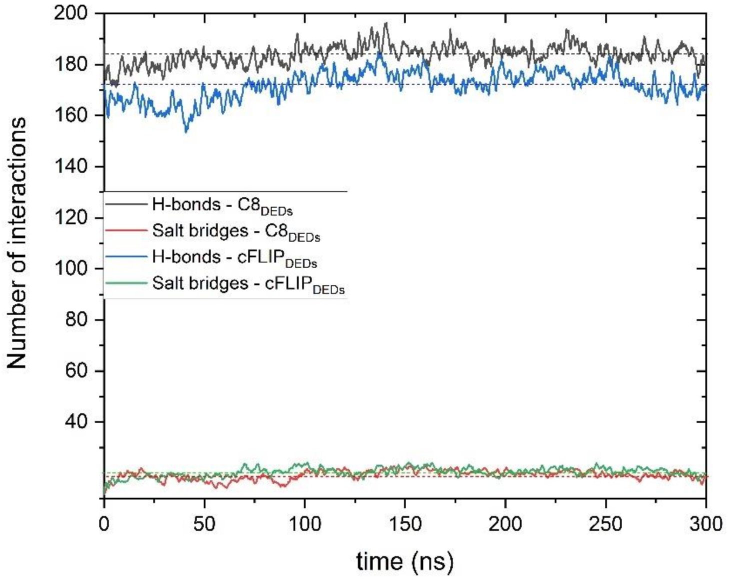

Figure S16. The number of hydrogen bond and salt bridge interactions within $C8_{DEDs}$ and $cFLIP_{DEDs}$ molecules during the 300 ns MD simulation.

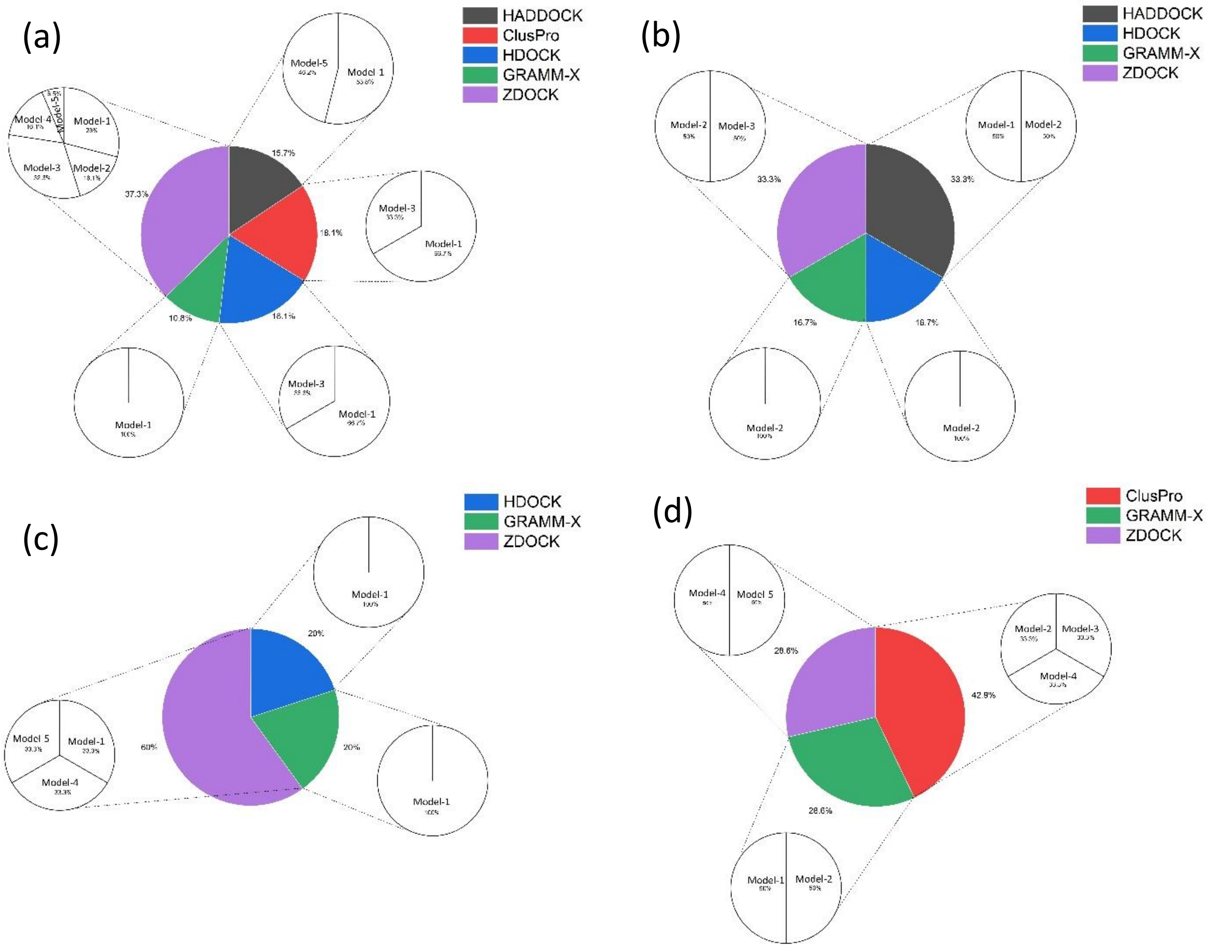

(e)

(f)

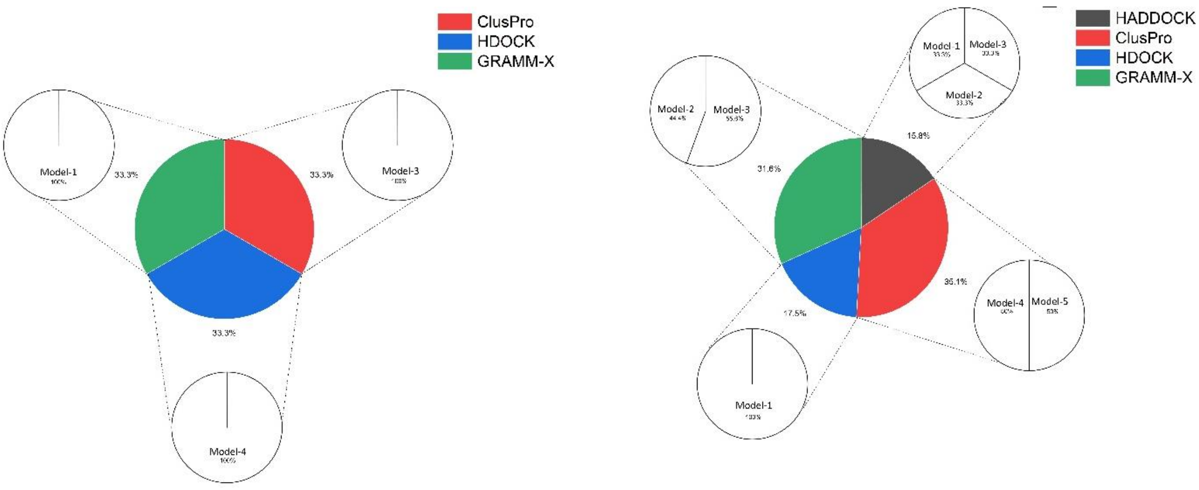

(g)

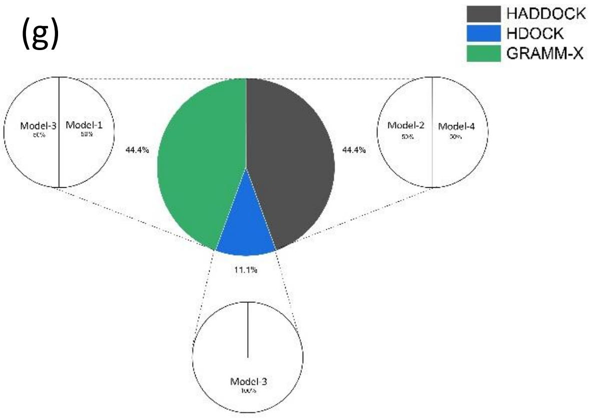

Figure S17. The contribution of each docking engines in the main clusters of (a) Fas_{DD}–Fas_{DD}, (b) $Fas_{1,DD}$–$FADD_{1,DD}$, (c) $Fas_{2,DD}$–$FADD_{2,DD}$, (d) $FADD_{DED}$–$C8_{DEDs}$, (e) $FADD_{DED}$–$cFLIP_{DEDs}$, (f) $C8_{DEDs}$–$C8_{DEDs}$, and (g) $C8_{DEDs}$–$cFLIP_{DEDs}$ complexes.

Table S4. The cluster component analysis for the main cluster of Fas_{DD}–Fas_{DD} complex.

Docking Engines	Models	Components	# of components
**ZDOCK**	ZDOCK_1	ZDOCK_1_1 to ZDOCK_1_10 except ZDOCK_1_4	9
	ZDOCK_2	ZDOCK_2_1 to ZDOCK_2_5	5
	ZDOCK_3	ZDOCK_3_1 to ZDOCK_3_10	10
	ZDOCK_4	ZDOCK_4_1 to ZDOCK_4_5	5
	ZDOCK_5	ZDOCK_5_1 and ZDOCK_5_2	2
**CLusPro**	CLusPro_1	CLusPro_1_1 to CLusPro_1_10	10
	CLusPro_3	CLusPro_3_1 to CLusPro_3_5	5
**HDOCK**	HDOCK_1	HDOCK_1_1 toHDOCK_1_10	10
	HDOCK_3	HDOCK_3_1 toHDOCK_3_5	5
**HADDOCK**	HADDOCK_1	HADDOCK_1_2 and HADDOCK_1_3 and HADDOCK_1_6 to HADDOCK_1_10	7
	HADDOCK_5	HADDOCK_5_2 and HADDOCK_5_6 to HADDOCK_5_10	6
**GRAMM-X**	GRAMM-X_1	GRAMM-X_1_2 to GRAMM-X_1_10	9

Table S5. The cluster component analysis for the main cluster of $Fas_{1,DD}$–$FADD_{1,DD}$ complex.

Docking Engines	Models	Components	# of components
**ZDOCK**	ZDOCK_2	ZDOCK_2_1 to ZDOCK_2_10	10
	ZDOCK_3	ZDOCK_3_1 to ZDOCK_3_10	10
**HADDOCK**	HADDOCK_1	HADDOCK_1_1 to HADDOCK_1_10	10
	HADDOCK_2	HADDOCK_2_1 to HADDOCK_2_10	10
**HDOCK**	HDOCK_2	HDOCK_2_1 to HDOCK_2_10	10
**GRAMM-X**	GRAMM-X_2	GRAMM-X_2_1 to GRAMM-X_2_10	10

Table S6. The cluster component analysis for the main cluster of $Fas_{2,DD}$–$FADD_{2,DD}$ complex.

Docking Engines	Models	Components	# of components
**ZDOCK**	ZDOCK_1	ZDOCK_1_1 to ZDOCK_1_10	10
	ZDOCK_4	ZDOCK_4_1 to ZDOCK_4_10	10
	ZDOCK_5	ZDOCK_5_1 to ZDOCK_5_10	10
**HDOCK**	HDOCK_1	HDOCK_1_1 to HDOCK_1_10	10
**GRAMM-X**	GRAMM-X_1	GRAMM-X_1_1 to GRAMM-X_1_10	10

Table S7. The cluster component analysis for the main cluster of $FADD_{DED}$–$C8_{DEDs}$ complex.

Docking Engines	Models	Components	# of components
**CLusPro**	CLusPro_2	CLusPro_2_1 to CLusPro_2_10	10
	CLusPro_3	CLusPro_3_1 to CLusPro_3_10	10
	CLusPro_4	CLusPro_4_1 to CLusPro_4_10	10
**GRAMM-X**	GRAMM-X _1	GRAMM-X _1_1 to GRAMM-X _1_10	10
	GRAMM-X _2	GRAMM-X _2_1 to GRAMM-X _2_10	10
**ZDOCK**	ZDOCK _4	ZDOCK _4_1 to ZDOCK _4_10	10
	ZDOCK _5	ZDOCK _5_1 to ZDOCK _5_10	10

Table S8. The cluster component analysis for the main cluster of $FADD_{DED}$–$cFLIP_{DEDs}$ complex.

Docking Engines	Models	Components	# of components
**CLusPro**	CLusPro_3	CLusPro_3_1 to CLusPro_3_10	10
**GRAMM-X**	GRAMM-X _1	GRAMM-X _1_1 to GRAMM-X _1_10	10
**HDOCK**	HDOCK _4	HDOCK _4_1 to HDOCK _4_10	10

Table S9. The cluster component analysis for the main cluster of $C8_{DEDs}$–$C8_{DEDs}$ complex.

Docking Engines	Models	Components	# of components
**CLusPro**	CLusPro_4	CLusPro_4_1 to CLusPro_4_10	10
	CLusPro_5	CLusPro_5_1 to CLusPro_5_10	10
**GRAMM-X**	GRAMM-X _2	GRAMM-X _2_1 to GRAMM-X _2_10	10
	GRAMM-X _3	GRAMM-X _3_1 to GRAMM-X _3_8	8
**HDOCK**	HDOCK _1	HDOCK _1_1 to HDOCK _1_10	10
**HADDOCK**	HADDOCK_1	HADDOCK_1_6 to HADDOCK_1_8	3
	HADDOCK_2	HADDOCK_2_1 to HADDOCK_2_3	3
	HADDOCK_3	HADDOCK_3_1 to HADDOCK_1_3	3

Table S10. The cluster component analysis for the main cluster of $C8_{DEDs}$–$cFLIP_{DEDs}$ complex.

Docking Engines	Models	Components	# of components
**HADDOCK**	HADDOCK_2	HADDOCK_2_1 to HADDOCK_2_10	10
	HADDOCK_4	HADDOCK_4_1 to HADDOCK_4_10	10
**GRAMM-X**	GRAMM-X _1	GRAMM-X _1_1 to GRAMM-X _1_10	10
	GRAMM-X _3	GRAMM-X _3_1 to GRAMM-X _3_10	10
**HDOCK**	HDOCK _3	HDOCK _3_1 to HDOCK _1_5	5

References

1. Dominguez, C.; Boelens, R.; Bonvin, A. M. J. J., HADDOCK: A Protein−Protein Docking Approach Based on Biochemical or Biophysical Information. *J. Am. Chem. Soc.* **2003**, 125, 1731-1737.

2. van Zundert, G. C. P.; Rodrigues, J. P. G. L. M.; Trellet, M.; Schmitz, C.; Kastritis, P. L.; Karaca, E.; Melquiond, A. S. J.; van Dijk, M.; de Vries, S. J.; Bonvin, A. M. J. J., The HADDOCK2.2 Web Server: User-Friendly Integrative Modeling of Biomolecular Complexes. *J. Mol. Biol.* **2016**, 428, 720-725.

3. Kozakov, D.; Hall, D. R.; Xia, B.; Porter, K. A.; Padhorny, D.; Yueh, C.; Beglov, D.; Vajda, S., The ClusPro web server for protein–protein docking. *Nat. Protoc.* **2017**, 12, 255-278.

4. Comeau, S. R.; Gatchell, D. W.; Vajda, S.; Camacho, C. J., ClusPro: an automated docking and discrimination method for the prediction of protein complexes. *Bioinformatics* **2004**, 20, 45-50.

5. Yan, Y.; Tao, H.; He, J.; Huang, S.-Y., The HDOCK server for integrated protein–protein docking. *Nat. Protoc.* **2020**, 15, 1829-1852.

6. Yan, Y.; Zhang, D.; Zhou, P.; Li, B.; Huang, S.-Y., HDOCK: a web server for protein–protein and protein–DNA/RNA docking based on a hybrid strategy. *Nucleic Acids Res.* **2017**, 45, W365-W373.

7. Tovchigrechko, A.; Vakser, I. A., GRAMM-X public web server for protein–protein docking. *Nucleic Acids Res.* **2006**, 34, W310-W314.

8. Chen, R.; Li, L.; Weng, Z., ZDOCK: An initial-stage protein-docking algorithm. *Proteins: Structure, Function, and Bioinformatics* **2003**, 52, 80-87.

9. Pierce, B. G.; Hourai, Y.; Weng, Z., Accelerating Protein Docking in ZDOCK Using an Advanced 3D Convolution Library. *PLoS One* **2011**, 6, e24657.
